# Test-retest reliability and validity of vagally-mediated heart rate variability to monitor internal training load in older adults: a within-subjects (repeated-measures) randomized study

**DOI:** 10.1186/s13102-024-00929-y

**Published:** 2024-06-27

**Authors:** Patrick Manser, Eling D. de Bruin

**Affiliations:** 1 Motor Control and Learning Group, Institute of Human Movement Sciences and Sport, Department of Health Sciences and Technology, ETH Zurich, Zurich, Switzerland; 2 Department of Health, OST - Eastern Swiss University of Applied Sciences, St. Gallen, Switzerland; 3 Division of Physiotherapy, Department of Neurobiology, Care Sciences and Society, Karolinska Institute, Stockholm, Sweden

**Keywords:** Autonomic nervous system, Biomarkers, Exercise, Exergaming, Neurosciences

## Abstract

**Background:**

Vagally-mediated heart rate variability (vm-HRV) shows promise as a biomarker of internal training load (ITL) during exergame-based training or motor-cognitive training in general. This study evaluated the test-retest reliability of vm-HRV during exergaming in healthy older adults (HOA) and its validity to monitor ITL.

**Methods:**

A within-subjects (repeated-measures) randomized study was conducted that included baseline assessments and 4 measurement sessions. Participants played 5 exergames at 3 standardized levels of external task demands (i.e., “easy”, “challenging”, and “excessive”) in random order for 90 s. Test-retest reliability was assessed on the basis of repeated-measures analyses of variance (ANOVA), intraclass correlation coefficients (ICC_3,1_), standard errors of measurement (SEM), and smallest detectable differences (SDD). Validity was determined by examining the effect of game level on vm-HRV in the ANOVA.

**Results:**

Fourty-three HOA (67.0 ± 7.0 years; 58.1% females (25 females, 18 males); body mass index = 23.7 ± 3.0 kg·m^−2^) were included. Mean R-R time intervals (mRR) and parasympathetic nervous system tone index (PNS-Index) exhibited mostly good to excellent relative test-retest reliability with no systematic error. Mean SEM% and SDD% were 36.4% and 100.7% for mRR, and 44.6% and 123.7% for PNS-Index, respectively. Significant differences in mRR and PNS-Index were observed between standardized levels of external task demands, with mostly large effect sizes (mean *r* = 0.847). These results persisted irrespective of the type of neurocognitive domain trained and when only motoric and cognitive demands were manipulated while physical intensity was kept constant. The remaining vm-HRV parameters showed inconsistent or poor reliability and validity.

**Conclusion:**

Only mRR and PNS-Index demonstrated reliable measurement and served as valid biomarkers for ITL during exergaming at a group level. Nonetheless, the presence of large SEMs hampers the detection of individual changes over time and suggests insufficient precision of these measurements at the individual level. Future research should further investigate the reliability and validity of vm-HRV with a specific focus on comparing different measurement methodologies and exercise conditions, particularly focusing on ultra-short-term HRV measurements, and investigate the potential implications (i.e., superiority to other markers of ITL or monitoring strategies?) of using vm-HRV as a biomarker of ITL.

**Supplementary Information:**

The online version contains supplementary material available at 10.1186/s13102-024-00929-y.

## Introduction

### Background

The growing need to identify and implement effective measures for the prevention of neurocognitive disorders [1] has led to the development of new approaches. While motor-cognitive training is recommended for the prevention of neurocognitive disorders by a collaborative international guideline [2], the utilization of technology to facilitate the implementation of motor-cognitive training, for instance through exergaming [3], is becoming increasingly popular. Exergames offer several advantages over conventional motor-cognitive training which promote their effectiveness and are, thus, currently considered a more promising training approach than conventional physical and/or cognitive training [4–6]. One of the key advantages of exergames is the ease of use of additional opportunities for individualized training through real-time adaptivity of task demands according to monitored parameters such as performance, measures of brain activity, or internal training load [7–9]. Nevertheless, recent systematic reviews have identified a paucity of systematic reporting and control of physical and, in particular, neurocognitive demands during exergaming in the majority of studies [10, 11]. Consequently, although training load monitoring has generally improved significantly over the past decades [12], multiple research groups have advocated that future research endeavors should prioritize the identification of reliable and valid parameters to monitor training load in order to enhance the effectiveness of physical training in general and exergame-based training in particular [10, 13–16].

The use of specific markers of “internal training load” (ITL) has been recommended to tailor exercises to the individual’s capabilities and performance [10, 16, 17]. ITL during exergaming is mainly influenced by neurocognitive task demands and the physical exercise intensity [18]. In light of this, it has been recommended that physical exercise intensity be prescribed and objectively monitored [10, 11], for example in accordance with the guidelines provided by the American College of Sports Medicine [10, 19]. Assessing and monitoring neurocognitive task demands is more complex, and there is a plethora of available measurement methods [13, 20]. For exergame-based training, the use of validated game metrics or subjective self-report measures has been recommended to prescribe and monitor the motoric and cognitive demands [10]. While self-report measures have been demonstrated to generally have high levels of validity in measuring cognitive load [21], they are prone to bias [13] and a single response to assess cognitive load after completing a training session or task may be insufficient, because neurocognitive task demands may change over time [13, 20, 22]. It is therefore of great importance to identify specific and time-sensitive physiological markers for ITL, as these temporal changes should be captured by a valid marker for ITL [17].

Vagally-mediated heart rate variability (vm-HRV) has been identified as a promising parameter for monitoring ITL during simultaneous motor-cognitive training, such as exergaming [13, 23]. The “neurovisceral integration” model [24] and its advancements [25, 26] posit that vm-HRV indexes the functional integrity of the central autonomic network (CAN). The CAN regulates physiological, emotional, and cognitive responses to environmental challenges [26], which is precisely what should be reflected by an optimal marker for ITL [17, 27]. Phasic vm-HRV responses have been shown to be moderated by physical and cognitive capabilities and exercise demands [23], are sensitive to neurocognitive demands related to cognitive and mental effort in older adults [13, 28–31], and have been found suitable in distinguishing between varying intensities and durations of physical exercise [32–34].

Nevertheless, further research is necessary to ascertain the suitability of monitoring phasic vm-HRV responses as a biomarker of ITL during exergaming [23]. In addition, the validity, reliability, sensitivity to change, and applicability of vm-HRV monitoring should be evaluated. This includes determining whether vm-HRV can capture ITL changes within reasonable timeframes for adapting task demands in real-time. However, there is currently insufficient evidence regarding the validity and reliability of vm-HRV measurements during physical exercise [35, 36] or simultaneous motor-cognitive training [23] as well as ultra-short term (< 5 min) recordings [37] in HOA. To ensure that observed changes in the variable of interest are attributable to real changes rather than measurement error, it is a prerequisite to assess the test-retest reliability before further exploring the validity of vm-HRV to monitor ITL during exergame-based motor-cognitive training.

### Objectives

The primary objective of this study was to evaluate the test-retest reliability of vm-HRV during exergame-based motor-cognitive training in relation to different exergame demands in HOA. As secondary objective, the validity of vm-HRV to monitor ITL during exergame-based motor-cognitive training was investigated for parameters with acceptable test-retest reliability.

## Materials and methods

### Study design

A within-subjects (repeated-measures) randomized study including HOA (≥ 60 years of age) was conducted. The study was reported according to the Guidelines for Reporting Reliability and Agreement Studies (GRRAS) [38] (Supplementary File 1) as well as the Strengthening the Reporting of Observational Studies in Epidemiology (STROBE) checklist for cross-sectional studies [39] (Supplementary File 2). All study procedures took place at ETH Hönggerberg (Zurich, Switzerland) and were led by investigators from our research team trained in the application of the measurement techniques and protocols. There were no changes to the methods or outcome measures after trial commencement.

After recruitment and providing written informed consent (section ‘Methods - Recruitment & consent procedure’), participants were screened on eligibility (section ‘Methods - Eligibility criteria’ and Table 1), and the measurements were scheduled for eligible participants. At the first scheduled appointment, baseline assessments were performed, and participants were familiarized with the exergame training system “Senso” (Dividat AG, Schindellegi, Switzerland) with software version 22.4.0-360-gf9df00d5b. The system’s pressure-sensitive platform uses 20 sensors to detect the position and timing of participants’ movements, allowing them to control virtual exergame scenarios displayed on a frontal screen. Each participant completed 1 standardized exercise session to familiarize themselves with the exergame scenarios by play-testing each game for 2 min.

**Table 1:** Description of all eligibility criteria

Inclusion criteria	Exclusion criteria
Participants fulfilling all the following inclusion criteria were eligible:	The presence of any of the following criteria led to exclusion:
• healthy (based on self-report) older adults (≥ 60 years) • ability to stand for at least 10 min without assistance • German speaking	• mobility impairments (i.e., gait, balance) that prevent from study participation • presence of neurological disorders (i.e., epilepsy, stroke, multiple sclerosis, Parkinson’s disease, brain tumors, or traumatic disorders of the nervous system) • presence of any other unstable or uncontrolled diseases (e.g., uncontrolled high blood pressure and progressing or terminal cancer)

The following 4 appointments included the experimental procedures that were scheduled to take place at approximately the same time of the day (± 2 h). To minimize the influence of transient confounding effects on HRV, all participants were instructed verbally and in writing to follow a normal sleep routine the day before the experiment, to avoid intense physical activities and alcohol consumption within 24 h before the measurements, and to refrain from coffee and caffeinated drinks as well as food consumption at least 2 h before the measurements [40]. No compensation was granted to the participants, but detailed feedback on individual performance as well as the study outcomes in general was provided at the end of the study.

### Recruitment & consent procedure

HOA were recruited between January 2021 and June 2021 in collaboration with healthcare institutions in the larger area of Zurich by handing out leaflets to interested persons. All potential participants were fully informed about the study by trained investigators from our research team by providing verbal explanations and an information sheet. After sufficient time for considerations (i.e., at least 24 h after handing out the study information sheet, but on average around one week), interested persons willing to take part in the study provided written informed consent, were screened on eligibility in an in-person meeting, and the study sessions were scheduled.

### Eligibility criteria

All eligibility criteria are detailed in Table 1.

### Experimental procedures

All experimental procedures were preceded by a resting-state measurement of heart rate (HR_rest_) and vm-HRV (section ‘Measurement of HR and vm-HRV’). To account for differing exergaming conditions and distinguish between the physical and neurocognitive demands of exergaming, we evaluated the study objectives in exergaming as ‘simultaneous-incorporated’ (i.e., physical and cognitive demands are linked, and both change as a function of game complexity; phase 1) and ‘simultaneous-additional’ (phase 2) motor-cognitive training [41]. More specifically, phase 2 was based on a methodological framework for the contribution of physical and neurocognitive (i.e., game-) demands during exergaming (section ‘Step 2: Development and Validation of Adaptation Loop’ and figure 1 of [42]). A stepping task was used separated from game demands to keep the physical intensity constant, whereas the game demands were then manipulated. Based on this framework, changes in the overall ITL can mainly be attributed to the game demands (motoric and cognitive demands) since the physical exercise intensity is kept constant [42]. Each experimental phase included 2 measurements performed within 2 weeks.

**Fig. 1:**
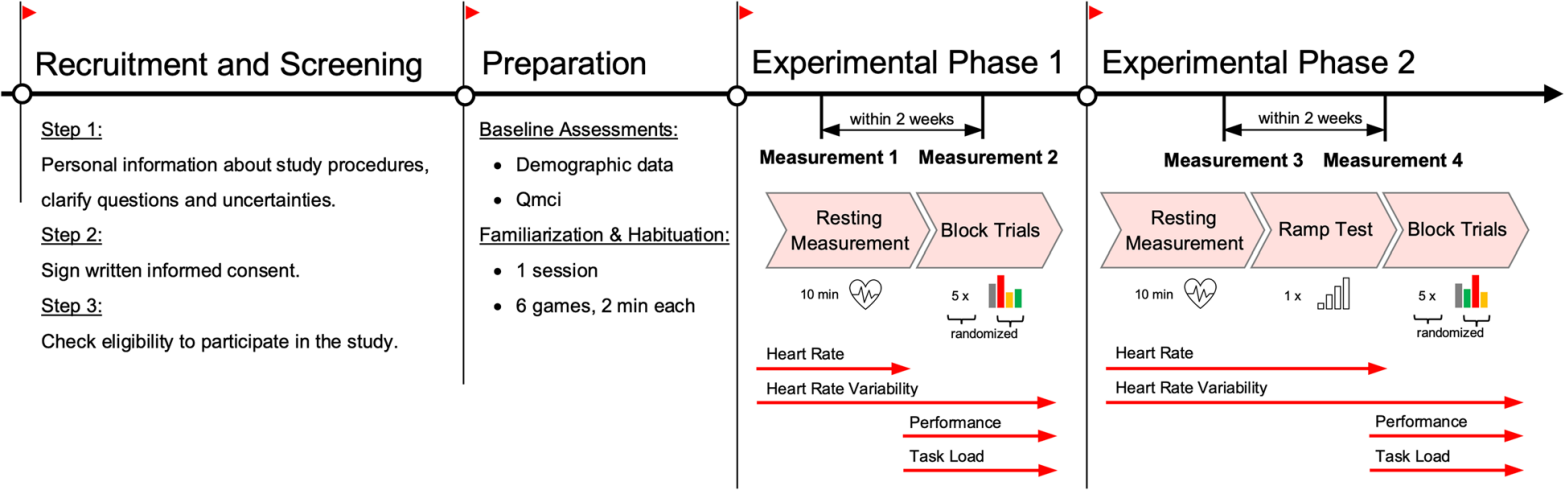
Overview of the study procedures. Color coding of the block trials referring to different levels of task demands: gray = adaptive, orange = easy, green = challenging, red = excessive. Abbreviations: Qmci, Quick mild cognitive impairment screen

The individual randomization of the order of games and levels of task demands was conducted by the outcome assessor, who used the randomization list from random.org. The same outcome assessor performed the test and retest measurements for individual participants. No blinding protocol was implemented. Participants were informed that different exergame demands would be applied, but were not given any information about the specific order of games and levels of task demands, nor the differences between them. Figure 1 summarizes the study procedures.

#### Experimental phase 1: evaluation as ‘simultaneous-incorporated’ motor-cognitive training

In phase 1, 5 exergames were performed in randomized order. To investigate our objectives in relation to different neurocognitive functions, 5 games that mainly demand attentional (‘Simple’), executive (‘Habitats’, ‘Targets’), and visuospatial (‘Tetris’) functions, as well as learning and memory (‘Simon’), were played (video demonstrations of the games see [43]). Four levels of external task demand (e.g., game type, task complexity, predictability of required tasks) were applied for each game (section ‘Standardized levels of external task demands’).

#### Experimental phase 2: evaluation as ‘simultaneous-additional’ motor-cognitive training

First, the minimal stepping frequency to reach a moderate level of physical intensity (i.e., ranging between 40 and 59% HRR [19]) was determined using a ramp test (start level = 80 steps/min, increases of 5 steps/min every 20 s until target HR (HR_target_) was reached). Participants followed the auditory rhythm of a metronome. Real-time HR measures (section ‘Measurement of HR and vm-HRV) were averaged over each 20 s—interval. HR_target_ was calculated on basis of the Karvonen method with a target intensity of 40% HRR: HR_target_ = (maximal HR (HR_max_) – HR_rest_) · 0.40 + HR_rest_ [44, 45] using the age-predicted HR_max_ = 208 – 0.7 · age.

During the block trials (Fig. 1), a variable amount of game demands was applied on top of this fixed physical intensity. The same 5 games and standardized levels of external task demands as in ‘Experimental Phase 1: Evaluation as ‘simultaneous-incorporated’ motor-cognitive training’ were used. To minimize the effect of fatigue due to physical exertion, participants rested until their HR was equal to their previous HR_rest_. Study investigators were instructed to maintain HR_rest_ within ± 5 bpm throughout the experimental session before starting a new exergame.

#### Standardized levels of external task demands

For each game, adaptive task demands were first applied to minimize learning effects. Subsequently, three standardized levels of external task demands (i.e., “easy”, “challenging”, “excessive”) were applied in randomized order for 90 s each. All external task demands were predetermined in consultation with a neuropsychologist experienced in exergame training with HOA. The aim of the 3 levels was to induce an underload in the easy condition, a challenging but feasible load in the challenging condition, and an overload in the excessive condition in an average HOA. The same external loads were applied for all study participants. In the adaptive task condition, the exergame demands were adjusted using “Senso’s” internal progression algorithm. This algorithm adjusts task demands in real time based on user performance, with the goal of providing optimal challenge. The game characteristics and the specific settings for all games and levels are described in Supplementary File 3.

### Outcomes & data analysis

#### Baseline assessments

Age, sex, body mass index (i.e., body weight [kg] / (height [m])^2^), physical activity behavior (i.e., measured as volume [min/week] of physical activity of at least moderate level), and self-reported intake of cardioactive medications (i.e., medications that have reported effects on HR and/or HRV; reported medications were categorized as cardioactive [yes / no] by agreement between the two authors) was assessed. Additionally, we screened the global level of cognitive functioning using the German Version [46] of the validated Quick Mild Cognitive Impairment Screen (Qmci) [47–50], which was administered and evaluated according to published guidelines [48].

#### Measurement of HR and vm-HRV

Resting HR and vm-HRV were measured while sitting in a comfortable position on a chair, without speaking, with both feet flat on the floor with knees at a 90° angle, hands on thighs (i.e., palms facing upward), and eyes closed [40]. Measurements were taken in a quiet room with dimmed lighting and at room temperature.

Multi-lead ECG is considered the gold standard for measuring HRV [51]. However, portable heart rate monitors (e.g., chest belts) are widely spread and have better ease of use for monitoring ITL during everyday training. Given the consistent evidence demonstrating a small amount of absolute error in HRV measurements obtained from the measurement of inter-beat-intervals through one-lead ECG via portable heart rate monitors when compared to multi-lead ECG recordings [36, 52], data was collected using a HR monitor (Polar M430) with sensor (Polar H10). The acclimatization phase was 5 min, followed by a 5 min resting measurement, the recommended duration for short-term recordings [40, 53]. The start of the resting measurement was not announced to participants [40]. In addition, R-R Intervals were continuously recorded throughout the experimental procedures.

For both, resting and on-task measurements, a sampling rate of 1000 Hz was used to provide a temporal resolution of 1 ms for each R–R interval [54]. R-R data was transmitted to Kubios HRV Premium (Kubios Oy, Kuopio, Finland, Version 3.4) for analysis. Kubios HRV is a scientifically validated software for HRV analysis and has achieved a gold-standard status in scientific research [55–58]. The automatic beat correction algorithm and noise handling provided by the software was used to correct for artifact and/or ectopic beats. The algorithm was validated for measurements at rest and was additionally tested for exercise measurements and provides reliable HRV analysis by reducing the effect of potential artefacts to a tolerable level [55]. The entire 5-min resting measurement was analyzed, while the last 60 s of on-task measurements were selected for analysis. After removing inter-beat-interval time series non-stationarities by detrending analysis using the smoothness priors method approach (settings: detrending method = smoothn priors, Lambda = 500, fc = 0.035 Hz), mean values of mainly vm-HRV indices were calculated for each segment. The mean R-R time interval (mRR), root mean square of successive RR interval differences (RMSSD), absolute power of the high-frequency (0.15–0.4 Hz; HF) band, relative power of HF (in normal units; HF [n.u.] = HF [ms^2^] / (total power [ms^2^] – very low frequency (0.00–0.04 Hz [ms^2^])), and the Poincaré plot standard deviation perpendicular to the line of identity (SD1) were considered [37, 40, 53, 59, 60]. Additionally, the parasympathetic nervous system tone index (PNS-Index) was calculated that compares PNS activity to normal resting values [60].

#### Assessment of perceived task load

Participants rated their subjective task load immediately after completing each game using the NASA Task Load Index (TLX). The NASA TLX consists of 6 rating scales (subjective effort, mental demand, temporal demand, physical demand, perception of performance and frustration) ranging between ‘0 = very low’ and ‘20 = very high’ [61]. The raw TLX (RTLX) was calculated by summing up all sub-scores without weighting [62].

#### Data management

Study investigators received comprehensive training on study procedures following the Guidelines of Good Clinical Practice (GCP) and detailed working instructions. The principal investigator oversaw methodological standards and ensured quality data collection using the Castor EDC data management system (Ciwit BV, Amsterdam, The Netherlands). Data entry in electronic case report forms (eCRFs) included pre-programmed range checks. A second study investigator cross-checked all data entries before exporting the data for analysis. To minimize bias, standardized measurement procedures and participant instructions were followed according to detailed work instructions for all outcome measures.

### Statistics

Statistical analyses were executed using R Version R 3.6.2 GUI 1.70 El Capitan build (7735) (© The R Foundation) in line with RStudio Version 1.2.5033 (RStudio, Inc.). First, descriptive statistics were computed for all outcome variables [63–65]. Normality distribution was checked using the Shapiro–Wilk test. The level of significance was set to p ≤ 0.05 (2-sided). Data was reported as mean ± standard deviation for data fulfilling all the assumptions that would subsequently justify parametric statistical analyses. In case these assumptions were not met, medians (interquartile ranges) were reported. All the following statistical procedures were performed for each experimental phase separately.

#### Test-retest reliability of vm-HRV

For a comprehensive assessment of test-retest reliability, a 3-level approach was adopted as recommended by Weir [66]. Only data from participants with complete datasets of high-quality data (i.e., less than 5% of beats corrected by the automatic beat correction algorithm of Kubios HRV Premium) were included in the analysis.

First, a 2-way repeated-measures analyses of variance (ANOVA; timepoints of measurement X standardized levels of external task demands) or (in case of non-parametric analyses) a robust ANOVA using the nparLD package [67] was computed to examine systematic error. In case of violation of the assumption of sphericity (assessed using Mauchly’s test), Greenhouse–Geisser corrections were applied [65].

Second, relative reliability was assessed by calculating intraclass correlation coefficients (ICC_3,1_) estimates and their 95% confidence intervals for the agreement between repeated measurements [66, 68, 69]. ICC’s were interpreted as representing poor (ICC_3,1_ < 0.50), fair (0.5 ≤ ICC_3,1_ < 0.75), good (0.75 ≤ ICC_3,1_ < 0.9), or excellent (ICC_3,1_ ≥ 0.9) agreement [68].

Third, absolute reliability was assessed by calculating standard errors of measurement (SEM) = standard error (of both measurements) * √(1 – *ICC*_3,1_); expressed as mean-normalized SEM (SEM%; SEM% = 100 * SEM / mean, combined mean value of both measurements) and the smallest detectable differences (SDD) = SEM * 1.96 *√2; expressed as mean-normalized SDD (SDD% = 100 * SDD / mean, combined mean value of both measurements) [66].

#### Validity of vm-HRV to monitor ITL

Only outcome measures with acceptable test-retest reliability (i.e., at least fair test-retest reliability (i.e., ICC_3,1_ ≥ 0.5) in all 3 levels of standardized task demands) were considered eligible for the investigation on validity, because it has been defined as a prerequisite to ascertain the test-retest reliability before further exploring the validity of vm-HRV to monitor ITL during exergame-based motor-cognitive training to ensure that observed changes in the variable of interest are attributable to real changes rather than measurement error. Validity was checked by assessing whether there was an effect of game level (i.e. ‘easy’ vs. ‘challenging’ vs. ‘excessive’) on vm-HRV in the 2-way repeated-measures ANOVA (section ‘Test-retest reliability of vm-HRV’). In case of a significant main effect of game level and no significant interaction effect, post-hoc tests were computed by calculating pairwise t-test with Bonferroni correction or a Wilcoxon signed-rank test in case of data violating assumptions for parametric analyses [70]. Effect sizes r were calculated for the pairwise comparisons [65, 71] and interpreted as small (0.1 ≤ *r* < 0.3), medium (0.3 ≤ *r* < 0.5) or large (*r* > 0.5) [70]. To verify that the predetermined levels of external task demands changed ITL (approximated by means of the NASA-TLX rating), the same statistics were computed for the NASA-TLX score.

### Sample size justification

Sample size justification was based on the estimation approach for determining sample size for estimating ICC’s of Borg et al. [72] derived from Bonnet [73]. The latter provides sample size requirements for estimating ICC’s with a desired precision [73] while incorporating Bonett’s correction factor. Considering the criterion for good test-retest reliability (ICC ≥ 0.75) as anticipated ICC and a desired width of the confidence interval of ≤ 0.3 with 50% probability of obtaining the desired precision, a minimum sample size of *n* = 38 was required at a 95% confidence interval.

## Results

### Recruitment and participant flow

**Fig. 2:**
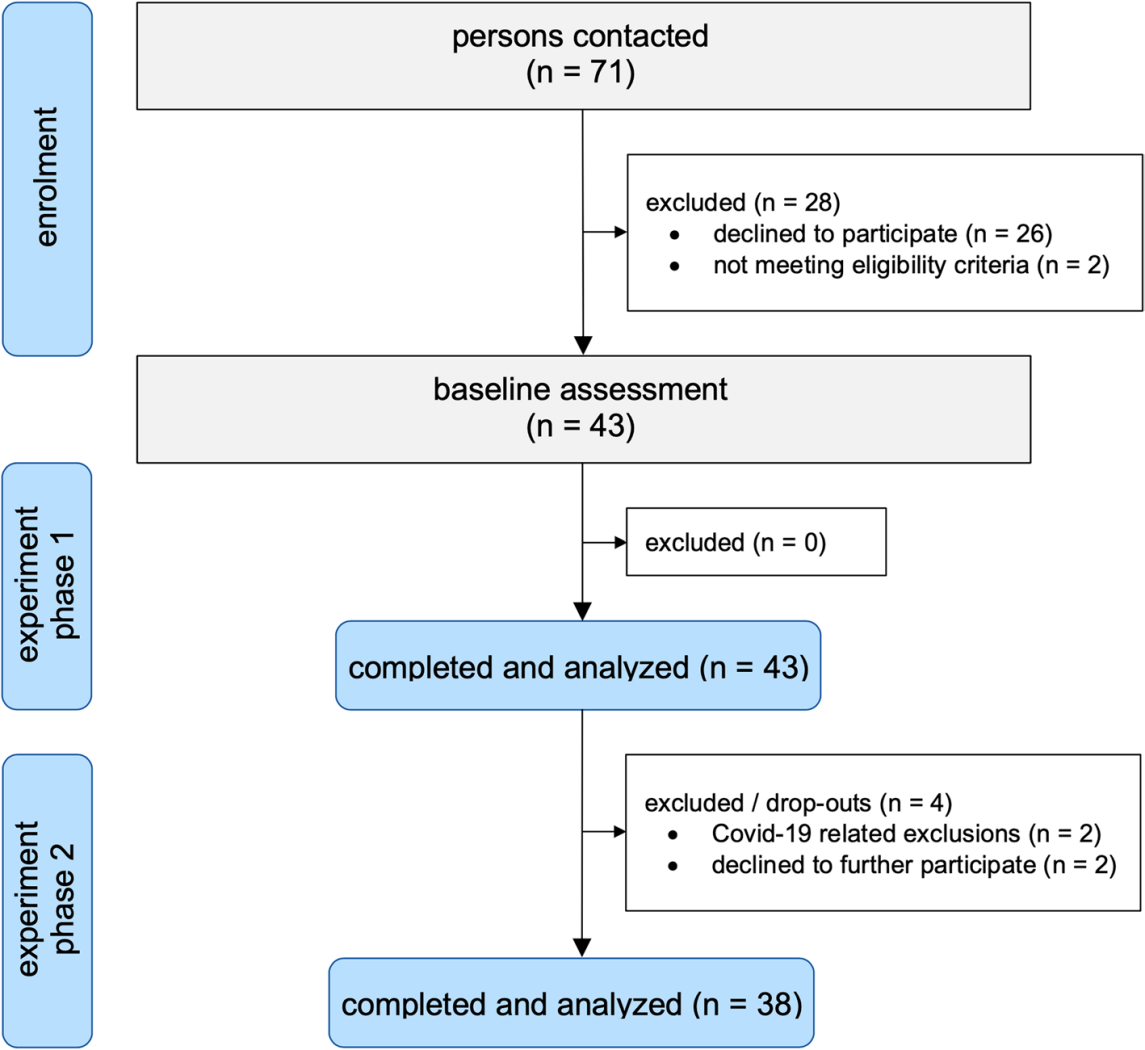
Summary of the participant flow throughout the study

A summary of the participant flow through the study is illustrated in Fig. 2.

Further recruitment was stopped when the planned minimum sample size of 38 participants completed the study.

### Baseline data and descriptive statistics

The baseline factors of the study participants are summarized in Table 2.

**Table 2:** Demographic characteristics of the study population

	Total Sample (*n* = 43)
Age [years]	67.0 (7.0)
Sex [% females]	58.1 (25 females, 18 males)
Body mass index [kg·m^−2^]	23.7 ± 3.0
Physical Activity [min ⋅ week^−1^]	330 (260)
Intake of cardioactive medication [% of participants]	34.9
Qmci total score	79.1 ± 8.5

Data is reported as mean ± standard deviation for data fulfilling all the assumptions that would subsequently justify parametric statistical analyses and median (interquartile range) for data violating these assumptions

*Abbreviations*: *Qmci* Quick mild cognitive impairment screen

The subjective ratings of task demands are summarized in Table 3. Significant (*p* < 0.001) differences with large effect sizes (mean *r* = 0.980, range = 0.513–1.229) were observed between all standardized levels of external task demands in both experimental phases, except for the game ‘Habitats’ level 2 vs. level 3 in experimental phase 1 and the game ‘Habitats’ level 1 vs. level 2 and level 2 vs. level 3 in experimental phase 2.

**Table 3:** Descriptive statistics and analysis of variance (ANOVA) for NASA-TLX total scores

**NASA-TLX total score**	**Experimental Phase 1**									**Experimental Phase 2**								
	**Descriptives**^**a**^	**Main Effect for Time**			**Main Effect for Level**					**Descriptives**^**a**^	**Main Effect for Time**			**Main Effect for Level**				
	mean ± SD or median (IQR)	*F*^b^	*p*^c^		*F*^d^	*p*^e^	post-hoc tests			mean ± SD or median (IQR)	*F*^b^	*p*^c^		*F*^d^	*p*^e^	post-hoc tests		
**Game: Simple**		0.3	0.601	^*n* =43^	130.5	< 0.001	comparison:	*p*-value:	effect size:		3.1	0.079	^*n* =35^	51.5	< 0.001	comparison:	*p*-value:	effect size:
Level 1	9.5 (19.0)						Level 1 vs. 2	< 0.001	0.909	37.5 (29.0)						Level 1 vs. 2	< 0.001	0.823
Level 2	16.0 (24.0)						Level 1 vs. 3	< 0.001	1.187	44.5 (35.0)						Level 1 vs. 3	< 0.001	1.164
Level 3	27.5 (26.5)						Level 2 vs. 3	< 0.001	1.133	57.5 (33.5)						Level 2 vs. 3	< 0.001	1.006
**Game: Targets**		10.3	0.001	^*n* =42^	132.0	< 0.001	comparison:	*p*-value:	effect size:		6.3	0.012	^*n* =34^	89.4	< 0.001	comparison:	*p*-value:	effect size:
Level 1	12.0 (21.0)						Level 1 vs. 2	< 0.001	1.168	30.0 (26.5)						Level 1 vs. 2	< 0.001	1.128
Level 2	29.0 (26.3)						Level 1 vs. 3	< 0.001	1.227	48.5 (28.5)						Level 1 vs. 3	< 0.001	1.229
Level 3	45.0 (31.8)						Level 2 vs. 3	< 0.001	1.194	64.0 (30.5)						Level 2 vs. 3	< 0.001	1.174
**Game: Tetris**		4.9	0.028	^*n* =42^	76.8	< 0.001	comparison:	*p*-value:	effect size:		16.3	< 0.001	^*n* =34^	56.0	< 0.001	comparison:	*p*-value:	effect size:
Level 1	19.0 (23.5)						Level 1 vs. 2	< 0.001	0.906	33.0 (29.3)						Level 1 vs. 2	< 0.001	0.994
Level 2	28.5 (40.3)						Level 1 vs. 3	< 0.001	1.189	45.0 (33.5)						Level 1 vs. 3	< 0.001	1.163
Level 3	58.5 (49.5)						Level 2 vs. 3	< 0.001	1.181	60.5 (32.3)						Level 2 vs. 3	< 0.001	0.917
**Game: Simon**		9.2	0.002	^*n* =41^	132.0	< 0.001	comparison:	*p*-value:	effect size:		12.2	< 0.001	^*n* =33^	68.4	< 0.001	comparison:	*p*-value:	effect size:
Level 1	15.0 (25.0)						Level 1 vs. 2	< 0.001	1.151	44.0 (36.0)						Level 1 vs. 2	< 0.001	0.910
Level 2	32.0 (32.5)						Level 1 vs. 3	< 0.001	1.227	52.0 (35.8)						Level 1 vs. 3	< 0.001	1.201
Level 3	60.0 (41.3)						Level 2 vs. 3	< 0.001	1.201	70.0 (32.5)						Level 2 vs. 3	< 0.001	1.162
**Game: Habitats**		0.0	0.936	^*n* =42^	4.9	0.013	comparison:	*p*-value:	effect size:		8.0	0.005	^*n* =35^	12.8	< 0.001	comparison:	*p*-value:	effect size:
Level 1	13.0 (25.0)						Level 1 vs. 2	< 0.001	0.513	31.0 (34.5)						Level 1 vs. 2	0.058	0.320
Level 2	16.5 (28.0)						Level 1 vs. 3	< 0.001	0.515	32.0 (37.0)						Level 1 vs. 3	< 0.001	0.813
Level 3	16.0 (31.3)						Level 2 vs. 3	0.218	0.190	36.5 (31.0)						Level 2 vs. 3	0.003	0.491

Presented are descriptive statistics, *F*-values, and *p*-values of the test and the retest data in Experimental Phase 1 and 2, respectively

*Abbreviations*: *HF* Absolute power of the high-frequency (0.15–0.4 Hz; HF) band, *HFnu* Relative power of HF (in normal units), *IQR* Interquartile range, *mRR* Mean R-R time interval, *n* Sample size, *NASA-TLX* NASA task load index, *RMSSD* Root mean square of successive RR interval differences, *SD1* Poincaré plot standard deviation perpendicular to the line of identity, *SD* Standard deviation

^a^Descriptive statistics data is reported as mean ± standard deviation for data fulfilling all the assumptions that would subsequently justify parametric statistical analyses and median (interquartile range) for data violating these assumptions

^b^*F*-value for the main effect of timepoint (test- vs. retest-measurement) from the two-way repeated measures ANOVA

^c^*P*-value for the main effect of timepoint (test- vs. retest-measurement) from the two-way repeated measures ANOVA

^d^*F*-value for the main effect of game level (Level 1 = easy, Level 2 = challenging, Level 3 = excessive) from the two-way repeated measures ANOVA

^e^*P*-value for the main effect of game level (Level 1 = easy, Level 2 = challenging, Level 3 = excessive) from the two-way repeated measures ANOVA

The mean stepping frequencies to achieve the target of ≥ 40% HRR were 149.5 ± 26.8 and 150.5 ± 25.5 at test and re-test measurement, respectively.

### Test-retest reliability of vm-HRV

Table 4 presents the results on test-retest reliability of vm-HRV. The parameters mRR and PNS-Index showed no systematic error and mostly good to excellent relative test-retest reliability (mean ICC_3,1_ = 0.855 (range = 0.434 to 0.939) and 0.787 (range = 0.519 to 0.903)), irrespective of the type of neurocognitive domain trained, experimental phase, or standardized level of external task demands. The mean SEM% and SDD% were 36.4% (range = 24.7 to 75.2) and 100.7% (range = 68.5 to 208.5) for the mRR, and 44.6% (range = 29.0 to 69.4) and 123.7% (range = 80.3 to 192.2) for the PNS-Index, respectively. The remaining vm-HRV parameters mostly showed no systematic error, but inconsistent or poor test-retest reliability.

**Table 4:**
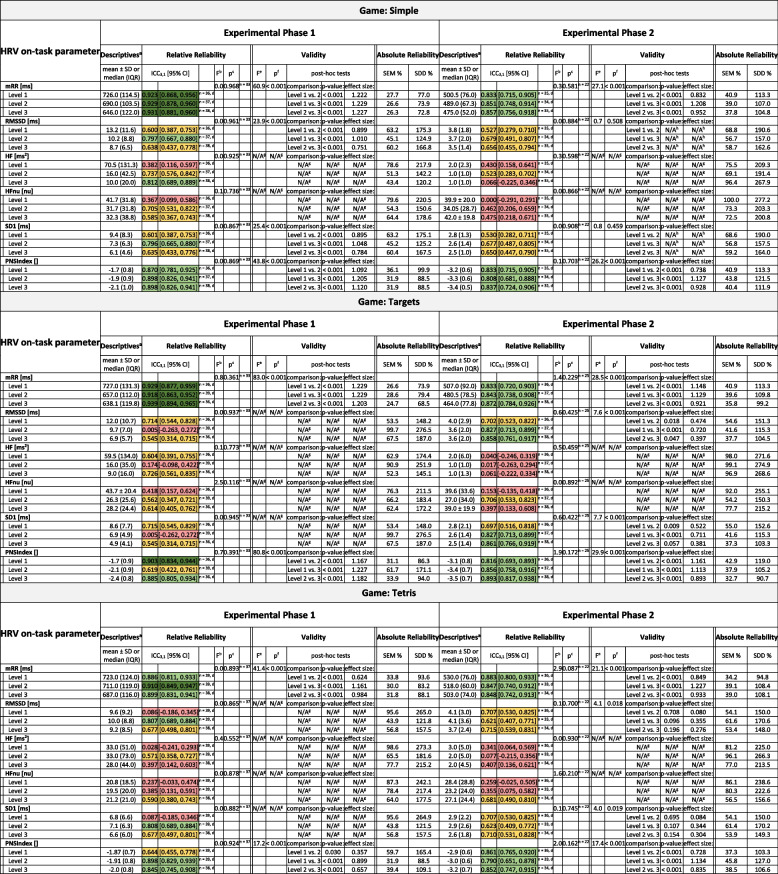

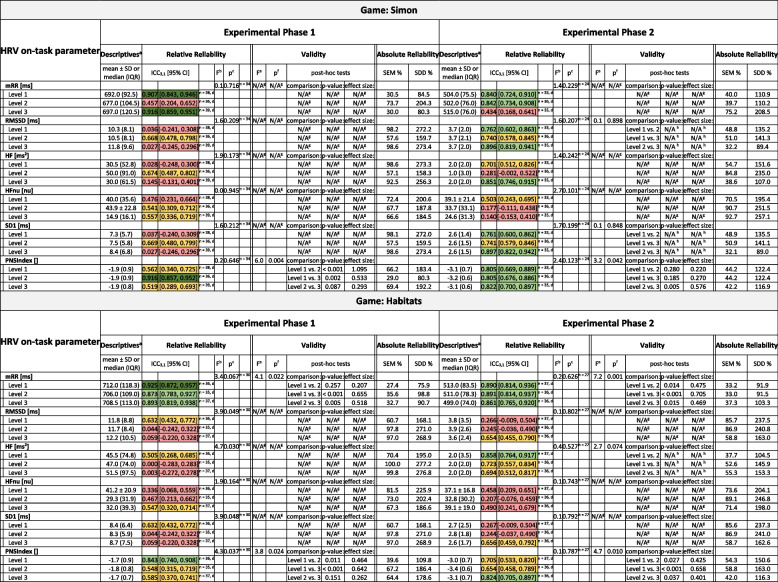
Test–retest reliability of HRV reactivity

Presented are descripitve statistics, ICCs, *F*-values, *p*-values, SEM%, and SDD%, of the test and the retest data in Experimental Phase 1 and 2, respectively

*Abbreviations*: *HF* Absolute power of the high-frequency (0.15–0.4 Hz; HF) band, *HFnu* Relative power of HF (in normal units), *ICC* Intraclass Correlation Coefficients, *IQR* Interquartile range, *mRR* Mean R-R time interval, *n* Sample size, *NASA-TLX* NASA task load index, *RMSSD* Root mean square of successive RR interval differences, *SD1* Poincaré plot standard deviation perpendicular to the line of identity, *SD* Standard deviation, *SDD* Smallest detectable difference, *SEM* Standard error of measurement

^a^Descriptive statistics; data is reported as mean ± standard deviation for data fulfilling all the assumptions that would subsequently justify parametric statistical analyses and median (interquartile range) for data violating these assumptions

^b^*F*-value for the main effect of timepoint (test- vs. retest-measurement) from the two-way repeated measures ANOVA

^c^*P*-value for the main effect of timepoint (test- vs. retest-measurement) from the two-way repeated measures ANOVA

^d^Missing data due to low quality data (≥ 5% of beats corrected by the automatic beat correction algorithm of Kubios HRV Premium) that was excluded from analysis

^e^*F*-value for the main effect of game level (Level 1 = easy, Level 2 = challenging, Level 3 = excessive) from the two-way repeated measures ANOVA

^f^*P*-value for the main effect of game level (Level 1 = easy, Level 2 = challenging, Level 3 = excessive) from the two-way repeated measures ANOVA

^g^Not applicable, because criteria for analysis were not met. In particular: Only outcome measures with at least a fair test-retest reliability (i.e., ICC3,1 ≥ 0.5) in all three levels of standardized task demands (i.e., Level 1 = easy, Level 2 = challenging, and Level 3 = excessive) were considered eligible for the exploration on validity

^h^Not applicable, because main effect is not significant = > no pairwise post-hoc comparisons

Colour coding for the interpretation of test-retest reliability:

### Validity of vm-HRV to monitor ITL

Table 4 presents the results on the validity of vm-HRV to monitor ITL. The parameters mRR and PNS-Index showed main effects for level with significant (*p* < 0.001) differences and with mostly large effect sizes (mean *r* = 0.847 (range = 0.207 to 1.229)) between all standardized levels of external task demands, irrespective of the type of neurocognitive domain trained and experimental phase. The remaining vm-HRV parameters showed varying results depending on the experimental phase and level of external task demand. In experimental phase 1, there was a main effect for level for the parameters RMSSD and SD1 in the game training attention (game = “Simple”), with significant (*p* < 0.001) differences and large effect sizes (ranging between 0.751 and 1.048) between all standardized levels of external task demands. In experimental phase 2 there was a main effect for level for the game training attention (game = “Simple”) as well as learning and memory (game = “Simon”), whereas there was no significant main effect for level for the game training visuospatial skills (game = “Tetris”), and inconsistent findings for the games training executive functions (games = “Targets” and “Habitats”).

## Discussion

This study evaluated the test-retest reliability of vm-HRV during exergame-based motor-cognitive training in relation to different exergame demands in HOA and its validity as a biomarker to monitor ITL. The results revealed the following key findings: The mRR and PNS-Index, measured with the Polar H10 sensor and calculated with Kubios HRV Premium, showed mostly good to excellent test-retest reliability without systematic error and consistent differences between the standardized levels of external task demands with mostly large effect sizes. These findings persisted irrespective of the type of neurocognitive domain trained and when only the game demands (motoric and cognitive demands) were manipulated while the physical intensity was kept constant at a moderate level. The remaining vm-HRV parameters showed inconsistent results or poor test-retest reliability and validity.

### Test-retest reliability of vm-HRV

To the best of our knowledge, there is only one comparable study that investigated test-retest reliability of HRV during physical or cognitive exercise in relation to different task demands in HOA. Mukherjee et al. [74] measured HRV with on-lead portable electrocardiogram (ECG) during 2 visual working memory tasks requiring different levels of mental effort (i.e., an “easy” and a “hard” trial) in 40 healthy older adults (age = 73.1 ± 4.9 years). They found high test-retest reliability for most HRV parameters, while time domain measures were the most reliable in both task conditions with Kendall’s τ ranging from 0.26 to 0.74 [74]. Another study by Guijt et al. [75] found good test-retest reliability during laboratory cycling at light exercise intensity in 26 healthy adults (age = 29.8 ± 8.5 years) in the parameters SDNN (ICC = 0.85 (0.70 – 0.93)) and RMSSD (ICC = 0.84 (0.67 – 0.92)), measured with a one-lead ECG via portable heart rate monitor.

Our results for raw data directly exported from Polar (mRR) and the PNS-Index are consistent with these findings. In contrast to the findings of these 2 studies, however, we had mixed findings for the reliability of remaining vm-HRV parameters. These inconsistent findings might be a result of the differing population characteristics (i.e., healthy adults analyzed in Guijt et al. [75] versus HOA analyzed in Mukherjee et al. [74] and this study) as well as measurement methodologies (i.e., different recording devices and durations that included Co2ntrol (Decon Medical Systems, Weesp, The Netherlands) [75], one-lead ECG with BioSemi (Bio- Semi, Amsterdam, The Netherlands) [74], or Polar HR monitor (Polar M430) and sensor (Polar H10) (this study) and durations of measurement of 10 min [75], 5 min [74], or 1 min (this study)) and conditions (i.e., different levels of physical and cognitive task demands as well as targeted neurocognitive domains (as defined in [76] in line with the Diagnostic and Statistical Manual of Mental Disorders 5th Edition (DSM-5) [77]) [37].

Regarding the measurement methodologies and conditions, Board et al. [36] systematically reviewed the literature and found excellent agreement of mRR measured with different Polar devices in different body positions at rest (ICCs between 0.94 and 1.00) as well as during exercise (ICCs between 0.93 (at vigorous exercise intensity) to 1.00 (at light exercise intensity) compared to multi-lead ECG. Additionally, they concluded that raw data on inter-beat interval time series derived from Polar heart rate monitors are valid for subsequent HRV analysis using validated Kubios HRV software [36], and that the calculated HRV parameters were found to be interchangeable when comparing values derived from the HR monitor-derived time series data with those derived from the multi-lead ECG data. [36]. Because we used the validated automatic beat correction algorithm and noise handling provided by Kubios HRV Premium [55], our mixed findings are likely to be primarily related to the short measurement duration and the high inter-individual variability of vm-HRV [78].

In contrast to Mukherjee et al. (2011) and Guijt et al. (2007), who used measurement durations of 5 min [74] and 10 min [75], we had a shorter measurement duration of only 1 min. Differences in contextual factors (such as age, health, recording methods, measurement conditions, artifacting procedures) may have greater impact on ultra-short-term measurements (< 5 min of measurement) than on longer recordings [37]. To check whether the inter-individual variability can be reduced, we repeated all analyses for vm-HRV reactivity (i.e., the absolute change from resting-state to on-task) [23], but did not find any relevant improvements compared to our original analyses (Supplementary File 4). Therefore, although it has been reported that measurements as short as 1 min may be sufficient to measure resting HR, SDNN and RMSSD [37], our data indicate that a measurement duration of 1 min may be too short for reliable measurement of RMSSD, HF, HFnu, and SD1 during exergame-based training or motor-cognitive training in general.

### Validity of vm-HRV to monitor ITL

To the best of our knowledge, this is the first study investigating phasic vm-HRV responses to exergaming. Nevertheless, our findings are consistent with recent literature regarding the sensitivity of vm-HRV to changes in task load of simultaneous motor-cognitive exercises and serious gaming in HOA. Hou et al. [79] analyzed HRV responses to serious games in HOA. They found significant decreases in vm-HRV during serious gaming, which differed between a cognitive aptitude game and reaction time games [79]. They replicated these findings in a consecutive study that also included patients with mild cognitive impairment. Significant decreases in vm-HRV were found during serious gaming, whereas the cognitive status of the study participants had no significant effect on the HRV [80]. This is consistent with the results of a systematic review that found significant withdrawal of vm-HRV in HOA during cognitive exercises, but contradicts the finding that vm-HRV in cognitive tasks is dependent on participant characteristics (i.e. level of cognitive functioning and physical fitness) [23].

Mukherjee et al. [74] found that time-domain measures of HRV (i.e. mRR, SDNN, and RMSSD) were the most sensitive to changes in mental task difficulty, with mostly medium to large effect sizes between an “easy” and a “hard” trial of visual working memory tasks. This is consistent with our findings as well as the literature reporting that vm-HRV is sensitive to neurocognitive demands (e.g., difficulty, complexity, duration) related to cognitive and mental effort in older adults [28–31]. Our findings are also consistent with the conceptual framework of Silvestrini et al. [81] and the *“vagal tank theory”* [27] suggesting that vm-HRV may indeed be a valid biomarker of ITL during (exergame-based) simultaneous motor-cognitive training. However, the SEMs were often very large, which hampers the detection of changes over time [66]. Because it is commonly accepted that the SEM is a fixed characteristic of any measure, regardless of the sample of study participants under investigation [66], this indicates insufficient precision of the individual measurements, which currently limits the applicability of vm-HRV to monitor ITL when measured with portable HR monitors (e.g. Polar H10).

### Implications for research

Although there is consistent evidence that HRV measurements obtained from the measurement of inter-beat-intervals through one-lead ECG via portable HR monitors shows a small amount of error compared to HRV derived from multi-lead ECG recordings [36, 52], further research is required to investigate the test-retest reliability of vm-HRV during exergame-based training and motor-cognitive training in general. In particular, future research should further investigate the reliability and validity of vm-HRV during exergame-based training and motor-cognitive training in general with a specific focus on comparing different measurement methodologies (e.g., measurement durations, technologies (i.e., measurement of inter-beat-intervals through one-lead ECG via portable heart rate monitor compared to multi-lead ECG recordings as well as different recording devices) as well as different analysis methodologies (e.g., beat correction and noise handling algorithms, or computation methods to calculated vm-HRV parameters), particularly focusing on ultra-short-term HRV measurements. Additionally, future research should more systemically evaluate the reliability and validity of vm-HRV under different exercise conditions (e.g., different levels of physical and/or cognitive task demands as well as targeted neurocognitive domains (e.g., as defined in [76] in line with the Diagnostic and Statistical Manual of Mental Disorders 5th Edition (DSM-5) [77]) and further examine the validity and potential implications of using vm-HRV as a biomarker of ITL during exergame-based training or motor-cognitive training in general. In particular, it should be investigated whether training that is prescribed and monitored according to real-time monitoring of ITL according to physiological parameters is superior to other markers for ITL and monitoring strategies, such as HRR, game metrics performance progression analysis or subjective ratings of ITL. These investigations have the potential to advance the utilization of vm-HRV in monitoring ITL during motor-cognitive training, and thereby pave the way to optimize individualized training prescription, reduce variability in training responses, and improve our understanding of the dose–response relationship between exercise and cognitive functioning [16].

### Limitations

The study has some key limitations that are worth mentioning. First, the measurement of vm-HRV was done using a one-lead ECG via portable HR monitor, and multi-lead ECG data was not collected in parallel to assess the agreement of the outcome measures with ECG data, although multi-lead ECG is considered the gold standard for measuring HRV [51]. This approach was chosen due to consistent evidence demonstrating a small amount of absolute error in HRV measurements obtained from the measurement of inter-beat-intervals through one-lead ECG via portable HR monitors when compared to multi-lead ECG recordings [36, 52]. Additionally, portable HR monitors (e.g., chest belts) are widely spread and have good ease of use for monitoring ITL during everyday training. Nevertheless, the use of data from multi-lead ECG recordings may provide more accurate measurements of vm-HRV compared to one-lead ECG via portable HR monitor due to a reduction of movement artifacts and the measurement of raw ECG signal instead of solely the inter-beat-intervals [40]. Second, despite designing and pilot-testing the study protocol to mitigate learning effects (i.e., by (1) the inclusion of a standardized familiarization session; (2) the commencement of each block with a trial with adaptive task demands before the three standardized levels of external task demands that were evaluated, (3) the randomization of the order of all games, as well as (4) the randomization of the three standardized levels of external task demands within each game), the data revealed the occurrence of some learning effects, as there were main effects of time in the perceived task load with a decrease in the perceived task load from the test to retest measurement in some exergaming-conditions. Third, while the study followed recommendations to minimize the influence of transient confounding effects [40], it was impossible to check whether the participants adhered to these instructions. This may have led to increased inter- and intra-individual variability of the vm-HRV measurements. Fourth, while we ensured that HR_rest_ remained within ± 5 bpm throughout the experimental session before starting a new exergame, we failed to do the same for vm-HRV. However, it has been reported that acute exercise effects post-exercise vm-HRV, which may be influenced by exercise intensity and/or duration [32, 82]. To mitigate the likelihood of any consequently biased outcomes, all standardized levels of external task demands as well as the five exergames were applied in randomized order. Finally, in the second experimental phase, vm-HRV values did not always reach a steady state for the last 60 s that were analyzed. This likely explains the mixed findings for test-retest reliability of vm-HRV during exergaming, as discussed in section ‘Test-retest reliability of vm-HRV’ and warrants future research to determine the minimum timeframe required to achieve steady state vm-HRV during exergaming in dependence on the physical and cognitive task demands.

## Conclusion

Only the vm-HRV parameters mRR and PNS-Index demonstrated reliable measurement and served as valid biomarkers for quantifying ITL during exergame-based motor-cognitive training at a group level. Nonetheless, the presence of large SEMs hampers the detection of individual changes over time and suggests insufficient precision of these measurements at the individual level. These findings emphasize the potential and current limitations of vm-HRV as a biomarker for monitoring ITL during exergame-based training or motor-cognitive training in general. Future research should further investigate the reliability and validity of vm-HRV with a specific focus on comparing different measurement methodologies and exercise conditions. This should include, but not be limited to, different measurement durations, measurement technologies, and analysis methodologies, as well as varying physical and cognitive tasks and task demands (more details see “Implications for research” section). Additionally, the potential implications of using vm-HRV as a biomarker of ITL during exergame-based training or motor-cognitive training in general should be examined. In particular, it should be investigated whether training prescribed and monitored according to real-time monitoring of ITL according to physiological parameters is superior to other markers for ITL and monitoring strategies, such as HRR, game metrics performance progression analysis, or subjective ratings of ITL. These investigations have the potential to advance the utilization of vm-HRV in monitoring ITL during motor-cognitive training, thereby paving the way to optimize individualized training prescriptions, reduce variability in training responses, and improve our understanding of the dose–response relationship between exercise and cognitive functioning [16].

### Supplementary Information

Supplementary Material 1.

## Data Availability

The datasets generated and/or analyzed during the current study are available in the Zenodo repository, 10.5281/zenodo.7824568.

## Abbreviations

ANOVA: Analysis of variance
CAN: Central autonomic network
ECG: Electrocardiogram
GCP: Good clinical practice
GRRAS: Guidelines for Reporting Reliability and Agreement Studies
HF: Absolute power of the high-frequency (0.15 – 0.4 Hz)
HF_nu_: Relative power of HF (in normal units; HF [n.u.] = HF [ms^2^] / (total power [ms^2^] – very low frequency (0.00 – 0.04 Hz [ms^2^]))
HOA: Healthy Older Adults
HR: Heart Rate
%HR_max_: Percentage of individual maximal heart rate
HR_max_: Maximal heart rate
HRR: Heart rate reserve
HR_rest_: Resting-state measurement of heart rate
HR_target_: Target heart rate
ICC: Intraclass Correlation Coefficient
ITL: Internal training load
mRR: Mean R-R time interval
PNS-Index: Parasympathetic nervous system tone index
Qmci: Quick Mild Cognitive Impairment Screen
RMSSD: Root mean square of successive RR interval differences
RTLX: Raw Task Load Index
SD1: Poincaré plot standard deviation perpendicular to the line of identity
SDD: Smallest detectable difference
SDD%: Mean-normalized smallest detectable difference
SEM: Standard error of measurement
SEM%: Mean-normalized standard error of measurement
TLX: Task Load Index
vm-HRV: Vagally-mediated Heart Rate Variability
