# Supplementary material for: Test-retest reliability and validity of vagally-mediated heart rate variability to monitor internal training load in older adults: a within-subjects (repeated-measures) randomized study
Source: BMC Sports Sci Med Rehabil. 2024 Jun 27;16:141. doi: 10.1186/s13102-024-00929-y (PMC11210148; doi:10.1186/s13102-024-00929-y)
Supplement: Supplementary file 1 — Supplementary Material 1. [file 13102_2024_929_MOESM1_ESM.pdf]

SUPPLEMENTARY FILES
Manser et al.

Test-Retest Reliability and Validity of vagally-mediated Heart Rate Variability to Monitor Internal Training Load in Older Adults: A within-subjects (repeated-measures) randomized study

Patrick Manser*[1] ORCID: 0000-0003-3300-6524 patrick.manser@hest.ethz.ch
Eling D. de Bruin[1-3] ORCID: 0000-0002-6542-7385 eling.debruin@hest.ethz.ch

[1] Motor Control and Learning Group - Institute of Human Movement Sciences and Sport, Department of Health Sciences and Technology, ETH Zurich, Zurich, Switzerland;
[2] Department of Health, OST - Eastern Swiss University of Applied Sciences, St. Gallen, Switzerland;
[3] Division of Physiotherapy, Department of Neurobiology, Care Sciences and Society, Karolinska Institute, Stockholm, Sweden

***Corresponding Author:**
Patrick Manser
ETH Zurich | Department of Health Sciences and Technology
Institute of Human Movement Sciences and Sport
Motor Control and Learning Group
HCP H24.3
Leopold-Ruzicka-Weg 4 | 8093 Zurich | Switzerland
Tel.: +41 79 519 96 46
Email: patrick.manser@hest.ethz.ch

Supplementary File 1 - Guidelines for Reporting Reliability and Agreement Studies (GRRAS) [1] Checklist

Table 1: Guidelines for Reporting Reliability and Agreement Studies (GRRAS) [1] Checklist

Section	Item #	Checklist item	Reported in section
Title/Abstract	1	Identify in title or abstract that inter-rater/intra-rater reliability or agreement was investigated.	'Title' as well as 'Abstract' (reported as "test-retest reliability", in accordance with [1])
Introduction	2	Name and describe the diagnostic or measurement device of interest explicitly.	'Introduction-Objectives'
	3	Specify the subject population of interest.	'Introduction-Objectives'
	4	Specify the rater population of interest (if applicable).	N/A
	5	Describe what is already known about reliability and agreement and provide a rationale for the study (if applicable).	'Introduction-Background'
Methods	6	Explain how the sample size was chosen. State the determined number of raters, subjects/objects, and replicate observations.	'Materials and Methods - Sample Size Justification'
	7	Describe the sampling method.	'Materials and Methods - Experimental Procedures' and 'Methods – Outcomes & Data Analysis - Measurement of HR and vm-HRV'
	8	Describe the measurement/rating process (e.g. time interval between repeated measurements, availability of clinical information, blinding).	'Materials and Methods - Experimental Procedures'
	9	State whether measurements/ratings were conducted independently.	'Materials and Methods - Experimental Procedures'
	10	Describe the statistical analysis.	'Materials and Methods – Statistics'
Results	11	State the actual number of raters and subjects/objects which were included and the number of replicate observations which were conducted.	'Results – Recruitment and participant flow', 'Results – Test-Retest Reliability of vm-HRV' (Table 4), and 'Results – Validity of of vm-HRV to monitor ITL' (Table 4)
	12	Describe the sample characteristics of raters and subjects (e.g. training, experience).	'Results – Recruitment and participant flow' (Table 2)
	13	Report estimates of reliability and agreement including measures of statistical uncertainty.	'Results – Test-Retest Reliability of vm-HRV' (Table 4), and 'Results – Validity of of vm-HRV to monitor ITL' (Table 4)
Discussion	14	Discuss the practical relevance of results.	'Discussion'
Auxiliary material	15	Provide detailed results if possible (e.g. online).	'Results – Test-Retest Reliability of vm-HRV' (Table 4), 'Results – Validity of of vm-HRV to monitor ITL' (Table 4), and Supplementary Files

Supplementary File 2 – Strengthening the Reporting of Observational Studies in Epidemiology (STROBE) [2] Checklist for cross-sectional studies

Table 2: Guidelines for Reporting Reliability and Agreement Studies (GRRAS) [1] Checklist

	Item No	Recommendation	Reported in section
**Title and abstract**	1	(a) Indicate the study's design with a commonly used term in the title or the abstract	'Title' as well as 'Abstract' (reported as "test-retest reliability", in accordance with [1])
		(b) Provide in the abstract an informative and balanced summary of what was done and what was found	'Abstract'
**Introduction**			
Background/rationale	2	Explain the scientific background and rationale for the investigation being reported	'Introduction-Background'
Objectives	3	State specific objectives, including any prespecified hypotheses	'Introduction-Objectives'
**Methods**			
Study design	4	Present key elements of study design early in the paper	'Materials and Methods – Study Design'
Setting	5	Describe the setting, locations, and relevant dates, including periods of recruitment, exposure, follow-up, and data collection	'Materials and Methods – Study Design' as well as 'Materials and Methods – Recruitment & Consent Procedure'
Participants	6	(a) Give the eligibility criteria, and the sources and methods of selection of participants	'Materials and Methods – Eligibility Criteria'
Variables	7	Clearly define all outcomes, exposures, predictors, potential confounders, and effect modifiers. Give diagnostic criteria, if applicable	'Materials and Methods – Experimental Procedures' as well as 'Materials and Methods – Outcomes & Data Analysis'
Data sources/ measurement	8*	For each variable of interest, give sources of data and details of methods of assessment (measurement). Describe comparability of assessment methods if there is more than one group	'Materials and Methods – Outcomes & Data Analysis'
Bias	9	Describe any efforts to address potential sources of bias	'Materials and Methods – Outcomes & Data Analysis'
Study size	10	Explain how the study size was arrived at	'Materials and Methods – ¨Sample Size Justification'
Quantitative variables	11	Explain how quantitative variables were handled in the analyses. If applicable, describe which groupings were chosen and why	'Materials and Methods – Outcomes & Data Analysis'
Statistical methods	12	(a) Describe all statistical methods, including those used to control for confounding	'Materials and Methods – Statistics'
		(b) Describe any methods used to examine subgroups and interactions	N/A
		(c) Explain how missing data were addressed	'Materials and Methods – Statistics'

		(d) If applicable, describe analytical methods taking account of sampling strategy	N/A
		(e) Describe any sensitivity analyses	N/A
**Results**			
Participants	13*	(a) Report numbers of individuals at each stage of study - e.g., numbers potentially eligible, examined for eligibility, confirmed eligible, included in the study, completing follow-up, and analyzed	'Results – Recruitment and participant flow'
		(b) Give reasons for non-participation at each stage	'Results – Recruitment and participant flow'
		(c) Consider use of a flow diagram	'Results – Recruitment and participant flow' – Figure 2
Descriptive data	14*	(a) Give characteristics of study participants (e.g., demographic, clinical, social) and information on exposures and potential confounders	'Results – Baseline Data and ´Descriptive Statistics'
		(b) Indicate number of participants with missing data for each variable of interest	'Results' - Table 3, Table 4
Outcome data	15*	Report numbers of outcome events or summary measures	'Results' - Table 3, Table 4
Main results	16	(a) Give unadjusted estimates and, if applicable, confounder-adjusted estimates and their precision (e.g., 95% confidence interval). Make clear which confounders were adjusted for and why they were included	'Results' - Table 3, Table 4
		(b) Report category boundaries when continuous variables were categorized	N/A
		(c) If relevant, consider translating estimates of relative risk into absolute risk for a meaningful time period	N/A
Other analyses	17	Report other analyses done - e.g., analyses of subgroups and interactions, and sensitivity analyses	N/A
**Discussion**			
Key results	18	Summarize key results with reference to study objectives	'Discussion'
Limitations	19	Discuss limitations of the study, taking into account sources of potential bias or imprecision. Discuss both direction and magnitude of any potential bias	'Discussion - Limitations'
Interpretation	20	Give a cautious overall interpretation of results considering objectives, limitations, multiplicity of analyses, results from similar studies, and other relevant evidence	'Discussion – Test-Retest Reliability of vm-HRV', as well as 'Discussion – Validity of vm-HRV to Monitor ITL'
Generalizability	21	Discuss the generalizability (external validity) of the study results	'Discussion – Test-Retest Reliability of vm-HRV', as well as 'Discussion – Validity of vm-HRV to Monitor ITL'
**Other information**			
Funding	22	Give the source of funding and the role of the funders for the present study and, if applicable, for the original study on which the present article is based	'Declarations – Roles and responsibilities – Funding'

*Give information separately for exposed and unexposed groups.

Note: An Explanation and Elaboration article discusses each checklist item and gives methodological background and published examples of transparent reporting. The STROBE

checklist is best used in conjunction with this article (freely available on the Web sites of PLoS Medicine at http://www.plosmedicine.org/, Annals of Internal Medicine at http://www.annals.org/, and Epidemiology at http://www.epidem.com/). Information on the STROBE Initiative is available at www.strobe-statement.org.

46 Supplementary File 3 - Game Characteristics and the Specific Settings for all Games
47 and Levels used

Game	Task Description	Task Demands				
		Parameter(s)	Condition & Settings			
			Level 1 = easy	Level 2 = challenging but feasible	Level 3 = excessive	adaptive
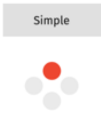	In the game "Simple", four circles are displayed in grey. As soon as one of the circles turns red, a step needs to be taken in the corresponding direction as fast as possible.	game speed (interstimulus-interval)<br>response window<br>predictability (order/time interval)<br>stepping direction(s)	6000 ms<br>3000 ms<br>Sequence 1: (↑ ← → ↓)<br>↑25 % → 25 % ← 25 % ↓25 %	3000 ms<br>1500 ms<br>Sequence 2: (↑ ← → ↓ ↓ ← → ↑)<br>↑25 % → 25 % ← 25 % ↓25 %	1500 ms<br>750 ms<br>random sequence<br>↑25 % → 25 % ← 25 % ↓25 %	adaptive task demands<br>start level: game speed (interstimulus-interval) = 3000 ms
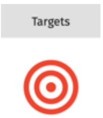	'Targets' requires the participant to hit incoming black balls in the middle of the target by stepping into the corresponding direction.	game speed (speed multiplayer)<br>stepping direction(s)	0.2<br>↑60 % → 20 % ← 20 % ↓0 %	0.45<br>↑40 % → 25 % ← 25 % ↓10 %	0.7<br>↑25 % → 25 % ← 25 % ↓25 %	adaptive task demands<br>start level: game speed (speed multiplier) = 0.45
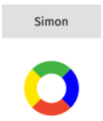	In the game 'Simon', a stepping sequence (i.e. indicated by a concurrent lighting up of a sequence of sections with different colors of a circle and a corresponding sound) has to be memorized and repeated by stepping into the corresponding direction.	sequence length<br>stepping direction(s)	2<br>↑50 % → 25 % ← 25 % ↓0 %	4<br>↑40 % → 25 % ← 25 % ↓10 %	6<br>↑25 % → 25 % ← 25 % ↓25 %	adaptive task demands<br>sequence length starts at 1 and prolongs with every successful completion. After a failure, sequency starts again at 1
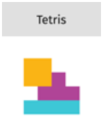	'Tetris' requires participants to rotate and move two-dimensional polygons (with varying shapes and colors) dropping one-by-one from top to the bottom. The aim of the game is to arrange complete rows of blocks to form solid horizontal lines, in order to let these lines disappear.	game speed (speed multiplier)	0.5	1	2	adaptive task demands<br>start level: game speed (speed multiplier) = 1
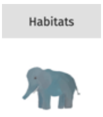	In the game "Habitats", animals move across the four landscapes in the picture. If an animal does not appear in its familiar surroundings, a step needs to be taken in this direction. However, animals shouldn't be disturbed in their natural habitat.	game speed (interstimulus-interval)<br>task complexity<br>stepping direction(s)	6000 ms<br>no inhibition tasks<br>↑60 % → 20 % ← 20 % ↓0 %	3000 ms<br>including inhibition tasks<br>↑40 % → 25 % ← 25 % ↓10 %	1500 ms<br>including inhibition tasks<br>↑25 % → 25 % ← 25 % ↓25 %	adaptive task demands<br>start level: game speed (interstimulus-interval) = 3000 ms

Supplementary File 4 – Additional Analysis of Test-Retest Reliability for vm-HRV reactivity (i.e., the absolute change from resting state to on-task)

Table 3 Test–Retest Reliability and Validity of HRV Reactivity

Game: Simple

Experiment Phase 1

HRV Parameter	Descriptives[a] mean ± SD / median (IQR)	Relative Reliability ICC$_{3,1}$ [95% CI]	F[b]	p[c]	Absolute Reliability SEM %	SDD %	Validity F[e]	p[f]	post-hoc tests		
mRR [ms]			0.1	0.817 n = 32			156.6	< 0.001	comparison:	p-value:	effect size:
Level 1	106.0 (85.0)	0.592 [0.376 , 0.748] n = 35, (d)			63.9	177.1			Level 1 vs. 2	< 0.001	1.222
Level 2	140.0 (105.0)	0.644 [0.446 , 0.782] n = 35, (d)			59.7	165.4			Level 1 vs. 3	< 0.001	1.229
Level 3	172.0 (131.3)	0.735 [0.573 , 0.841] n = 35, (d)			51.5	142.7			Level 2 vs. 3	< 0.001	1.228
RMSSD [ms]			1.5	0.225 n = 32			N/A [g]	N/A [g]	comparison:	p-value:	effect size:
Level 1	0.4 (8.7)	0.042 [-0.239 , 0.317] n = 35, (d)			97.9	271.3			N/A [g]	N/A [g]	N/A [g]
Level 2	2.5 (6.7)	0.328 [0.054 , 0.555] n = 35, (d)			82.0	227.2			N/A [g]	N/A [g]	N/A [g]
Level 3	4.6 (8.9)	0.303 [0.027 , 0.536] n = 35, (d)			83.5	231.4			N/A [g]	N/A [g]	N/A [g]
HF [ms^2]			1.7	0.186 n = 32			N/A [g]	N/A [g]	comparison:	p-value:	effect size:
Level 1	3 (105)	0.247 [-0.034 , 0.492] n = 35, (d)			86.8	240.5			N/A [g]	N/A [g]	N/A [g]
Level 2	21.5 (64.8)	0.016 [-0.264 , 0.293] n = 35, (d)			99.2	275.0			N/A [g]	N/A [g]	N/A [g]
Level 3	31.5 (80.0)	0.061 [-0.221 , 0.334] n = 35, (d)			96.9	268.6			N/A [g]	N/A [g]	N/A [g]
HFnu [nu]			0.0	0.947 n = 32			N/A [g]	N/A [g]	comparison:	p-value:	effect size:
Level 1	12.1 ± 26.2	0.216 [-0.066 , 0.467] n = 35, (d)			88.5	245.4			N/A [g]	N/A [g]	N/A [g]
Level 2	1.1 (27.8)	0.194 [-0.089 , 0.449] n = 35, (d)			89.8	248.9			N/A [g]	N/A [g]	N/A [g]
Level 3	2.8 ± 24.4	0.324 [0.049 , 0.552] n = 35, (d)			82.2	227.9			N/A [g]	N/A [g]	N/A [g]
SD1 [ms]			0.7	0.387 n = 32			N/A [g]	N/A [g]	comparison:	p-value:	effect size:
Level 1	0.2 (6.3)	0.013 [-0.266 , 0.291] n = 35, (d)			99.3	275.4			N/A [g]	N/A [g]	N/A [g]
Level 2	1.7 (4.7)	0.342 [0.070 , 0.566] n = 35, (d)			81.1	224.8			N/A [g]	N/A [g]	N/A [g]
Level 3	3.4 (6.2)	0.296 [0.019 , 0.531] n = 35, (d)			83.9	232.6			N/A [g]	N/A [g]	N/A [g]
PNSIndex []			0.0	0.968 n = 32			69.3	< 0.001	comparison:	p-value:	effect size:
Level 1	0.5 (0.6)	0.549 [0.319 , 0.718] n = 35, (d)			67.2	186.1			Level 1 vs. 2	< 0.001	1.087
Level 2	0.7 (0.6)	0.522 [0.285 , 0.699] n = 35, (d)			69.1	191.6			Level 1 vs. 3	< 0.001	1.212
Level 3	0.9 (0.9)	0.626 [0.421 , 0.771] n = 35, (d)			61.2	169.5			Level 2 vs. 3	< 0.001	1.147

Experiment Phase 2

HRV Parameter	Descriptives[a] mean ± SD / median (IQR)	Relative Reliability ICC$_{3,1}$[b] 95% CI	F[c]	p[d]	Validity F[e]	p[f]	post-hoc tests			Absolute Reliability SEM %	SDD %
mRR [ms]			1.1	0.287 n = 22	23.6	< 0.001	comparison:	p-value:	effect size:		
Level 1	302.0 (139.0)	0.834 [0.718 , 0.906] n = 32, (d)					Level 1 vs. 2	< 0.001	0.832	40.7	112.9
Level 2	310.5 (139.5)	0.819 [0.698 , 0.894] n = 34, (d)					Level 1 vs. 3	< 0.001	1.208	42.5	117.9
Level 3	269.5 (168.5)	0.869 [0.775 , 0.925] n = 33, (d)					Level 2 vs. 3	< 0.001	0.952	36.2	100.3
RMSSD [ms]			1.1	0.294 n = 22	0.4	0.7	comparison:	p-value:	effect size:		
Level 1	8.7 (9.0)	0.744 [0.578 , 0.851] n = 32, (d)					Level 1 vs. 2	N/A [h]	N/A [h]	50.1	140.2
Level 2	9.7 (11.5)	0.765 [0.615 , 0.861] n = 34, (d)					Level 1 vs. 3	N/A [h]	N/A [h]	48.5	134.4
Level 3	5.7 (13.0)	0.774 [0.626 , 0.868] n = 33, (d)					Level 2 vs. 3	N/A [h]	N/A [h]	47.5	131.8
HF [ms^2]			2.3	0.131 n = 22	0.4	0.7	comparison:	p-value:	effect size:		
Level 1	62.0 (92.0)	0.695 [0.506 , 0.820] n = 32, (d)					Level 1 vs. 2	N/A [h]	N/A [h]	55.2	153.1
Level 2	71.5 (123.5)	0.589 [0.368 , 0.747] n = 34, (d)					Level 1 vs. 3	N/A [h]	N/A [h]	64.1	177.7
Level 3	37.5 (104.5)	0.598 [0.375 , 0.755] n = 33, (d)					Level 2 vs. 3	N/A [h]	N/A [h]	63.4	175.7
HFnu [nu]			0.0	0.885 n = 22	N/A [g]	N/A [g]	comparison:	p-value:	effect size:		
Level 1	8.1 ± 32.2	0.200 [-0.097 , 0.464] n = 32, (d)					N/A [g]	N/A [g]	N/A [g]	89.4	247.9
Level 2	4.4 ± 31.8	0.363 [0.089 , 0.585] n = 34, (d)					N/A [g]	N/A [g]	N/A [g]	79.8	221.2
Level 3	5.3 ± 28.2	0.313 [0.029 , 0.550] n = 33, (d)					N/A [g]	N/A [g]	N/A [g]	82.9	229.7
SD1 [ms]			1.0	0.319 n = 22	0.2	0.8	comparison:	p-value:	effect size:		
Level 1	6.2 (6.3)	0.745 [0.579 , 0.851] n = 32, (d)					Level 1 vs. 2	N/A [h]	N/A [h]	50.5	140.0
Level 2	6.8 (7.8)	0.767 [0.618 , 0.863] n = 34, (d)					Level 1 vs. 3	N/A [h]	N/A [h]	48.3	133.8
Level 3	4.2 (9.1)	0.779 [0.634 , 0.871] n = 33, (d)					Level 2 vs. 3	N/A [h]	N/A [h]	47.0	130.3
PNSIndex []			0.3	0.607 n = 22	20.0	< 0.001	comparison:	p-value:	effect size:		
Level 1	1.8 (1.0)	0.799 [0.661 , 0.884] n = 32, (d)					Level 1 vs. 2	< 0.001	0.735	44.8	124.3
Level 2	1.9 (1.1)	0.728 [0.561 , 0.838] n = 34, (d)					Level 1 vs. 3	< 0.001	1.126	52.2	144.6
Level 3	1.5 (1.1)	0.802 [0.669 , 0.885] n = 33, (d)					Level 2 vs. 3	< 0.001	0.928	44.5	123.3

Continued

Game: Targets

Experiment Phase 1

HRV Parameter	Descriptives[a] mean ± SD / median (IQR)	Relative Reliability ICC$_{3,1}$ [95% CI]	F[b]	p[c]	Absolute Reliability SEM %	SDD %	Validity F[e]	p[f]	post-hoc tests		
mRR [ms]			0.8	0.364 n = 32			136.6	< 0.001	comparison:	p-value:	effect size:
Level 1	118.0 (83.0)	0.665 [0.474 , 0.796] n = 35, (d)			57.9	160.4			Level 1 vs. 2	< 0.001	1.229
Level 2	178.2 ± 70.8	0.689 [0.517 , 0.808] n = 38, (d)			55.8	154.6			Level 1 vs. 3	< 0.001	1.229
Level 3	224.4 ± 84.8	0.800 [0.671 , 0.882] n = 35, (d)			44.7	124.0			Level 2 vs. 3	< 0.001	1.203
RMSSD [ms]			0.5	0.498 n = 32			N/A [g]	N/A [g]	comparison:	p-value:	effect size:
Level 1	0.3 (7.1)	0.213 [-0.069 , 0.464] n = 35, (d)			88.7	245.9			N/A [g]	N/A [g]	N/A [g]
Level 2	3.2 (7.9)	0.073 [-0.198 , 0.334] n = 38, (d)			96.3	266.9			N/A [g]	N/A [g]	N/A [g]
Level 3	5.4 (9.3)	0.269 [-0.011 , 0.509] n = 35, (d)			85.5	237.0			N/A [g]	N/A [g]	N/A [g]
HF [ms^2]			0.2	0.632 n = 32			N/A [g]	N/A [g]	comparison:	p-value:	effect size:
Level 1	9.0 (74.0)	0.108 [-0.176 , 0.375] n = 35, (d)			94.4	261.8			N/A [g]	N/A [g]	N/A [g]
Level 2	22.0 (84.0)	0.005 [-0.263 , 0.272] n = 38, (d)			99.7	276.5			N/A [g]	N/A [g]	N/A [g]
Level 3	31.0 (73.5)	0.173 [-0.111 , 0.431] n = 35, (d)			90.9	252.1			N/A [g]	N/A [g]	N/A [g]
HFnu [nu]			0.2	0.658 n = 32			N/A [g]	N/A [g]	comparison:	p-value:	effect size:
Level 1	10.1 ± 21.8	0.281 [0.003 , 0.519] n = 35, (d)			84.8	235.0			N/A [g]	N/A [g]	N/A [g]
Level 2	4.0 ± 23.4	0.270 [0.003 , 0.501] n = 38, (d)			85.4	236.8			N/A [g]	N/A [g]	N/A [g]
Level 3	2.1 ± 24.9	0.296 [0.020 , 0.531] n = 35, (d)			83.9	232.6			N/A [g]	N/A [g]	N/A [g]
SD1 [ms]			0.2	0.627 n = 32			N/A [g]	N/A [g]	comparison:	p-value:	effect size:
Level 1	0.2 (5.1)	0.221 [-0.061 , 0.471] n = 35, (d)			88.3	244.6			N/A [g]	N/A [g]	N/A [g]
Level 2	2.2 (5.5)	0.080 [-0.191 , 0.340] n = 38, (d)			95.9	265.9			N/A [g]	N/A [g]	N/A [g]
Level 3	3.8 (6.6	0.267 [-0.012 , 0.508] n = 35, (d)			85.6	237.3			N/A [g]	N/A [g]	N/A [g]
PNSIndex []			0.1	0.728 n = 32			101.9	< 0.001	comparison:	p-value:	effect size:
Level 1	0.6 (0.6)	0.608 [0.397 , 0.758] n = 35, (d)			62.6	173.5			Level 1 vs. 2	< 0.001	1.177
Level 2	0.9 (0.7)	0.506 [0.276 , 0.681] n = 38, (d)			70.3	194.8			Level 1 vs. 3	< 0.001	1.225
Level 3	1.2 ± 0.6	0.702 [0.526 , 0.820] n = 35, (d)			54.6	151.3			Level 2 vs. 3	< 0.001	1.180

Experiment Phase 2

HRV Parameter	Descriptives[a] mean ± SD / median (IQR)	Relative Reliability ICC$_{3,1}$[b] 95% CI	F[c]	p[d]	Validity F[e]	p[f]	post-hoc tests			Absolute Reliability SEM %	SDD %
mRR [ms]			0.5	0.488 n = 24	40.5	< 0.001	comparison:	p-value:	effect size:		
Level 1	296.0 (155.5)	0.862 [0.767 , 0.921] n = 34, (d)					Level 1 vs. 2	< 0.001	1.141	37.1	103.0
Level 2	328.0 (160.8)	0.842 [0.737 , 0.908] n = 35, (d)					Level 1 vs. 3	< 0.001	1.124	39.7	110.2
Level 3	280.0 (155.3)	0.806 [0.681 , 0.886] n = 35, (d)					Level 2 vs. 3	< 0.001	0.926	44.0	122.1
RMSSD [ms]			0.0	0.830 n = 24	N/A [g]	N/A [g]	comparison:	p-value:	effect size:		
Level 1	8.8 (10.8)	0.494 [0.246 , 0.681] n = 34, (d)					N/A [g]	N/A [g]	N/A [g]	71.1	197.2
Level 2	9.7 (12.5)	0.529 [0.294 , 0.704] n = 35, (d)					N/A [g]	N/A [g]	N/A [g]	68.6	190.2
Level 3	6.8 (12.8)	0.576 [0.355 , 0.737] n = 35, (d)					N/A [g]	N/A [g]	N/A [g]	65.1	180.5
HF [ms^2]			0.0	1.000 n = 24	N/A [g]	N/A [g]	comparison:	p-value:	effect size:		
Level 1	60.5 (119.8)	0.522 [0.280 , 0.701] n = 34, (d)					N/A [g]	N/A [g]	N/A [g]	69.1	191.6
Level 2	72.5 (124.5)	0.450 [0.195 , 0.647] n = 35, (d)					N/A [g]	N/A [g]	N/A [g]	74.2	205.6
Level 3	46.5 (118.3)	0.455 [0.202 , 0.651] n = 35, (d)					N/A [g]	N/A [g]	N/A [g]	73.8	204.6
HFnu [nu]			0.5	0.482 n = 24	N/A [g]	N/A [g]	comparison:	p-value:	effect size:		
Level 1	9.4 ± 32.4	0.144 [-0.144 , 0.410] n = 34, (d)					N/A [g]	N/A [g]	N/A [g]	92.5	256.5
Level 2	1.4 ± 32.5	0.481 [0.234 , 0.670] n = 35, (d)					N/A [g]	N/A [g]	N/A [g]	72.0	199.7
Level 3	0.8 ± 28.6	0.203 [-0.080 , 0.455] n = 35, (d)					N/A [g]	N/A [g]	N/A [g]	89.3	247.5
SD1 [ms]			0.0	0.876 n = 24	N/A [g]	N/A [g]	comparison:	p-value:	effect size:		
Level 1	6.2 (7.5)	0.483 [0.233 , 0.674] n = 34, (d)					N/A [g]	N/A [g]	N/A [g]	71.9	199.3
Level 2	6.9 (8.8)	0.534 [0.300 , 0.707] n = 35, (d)					N/A [g]	N/A [g]	N/A [g]	68.3	189.2
Level 3	4.9 (9.0)	0.582 [0.362 , 0.740] n = 35, (d)					N/A [g]	N/A [g]	N/A [g]	64.7	179.2
PNSIndex []			0.3	0.616 n = 24	45.5	< 0.001	comparison:	p-value:	effect size:		
Level 1	1.9 (1.0)	0.761 [0.610 , 0.859] n = 34, (d)					Level 1 vs. 2	< 0.001	1.158	48.9	135.5
Level 2	2.0 (1.2)	0.744 [0.587 , 0.847] n = 35, (d)					Level 1 vs. 3	< 0.001	1.110	50.6	140.2
Level 3	1.6 (1.2)	0.729 [0.566 , 0.838] n = 35, (d)					Level 2 vs. 3	< 0.001	0.883	52.1	144.3

Continued

Game: Tetris

Experiment Phase 1

HRV Parameter	Descriptives[a] mean ± SD / median (IQR)	Relative Reliability ICC$_{3,1}$ [95% CI]	F[b]	p[c]	Absolute Reliability SEM %	SDD %	Validity F[e]	p[f]	post-hoc tests		
mRR [ms]			1.4	0.241 n = 36			46.6	< 0.001	comparison:	p-value:	effect size:
Level 1	114.0 (90.8)	0.543 [0.323 , 0.708] n = 38, (d)			67.6	187.4			Level 1 vs. 2	< 0.001	0.638
Level 2	132.1 ± 55.8	0.551 [0.333 , 0.713] n = 38, (d)			67.0	185.7			Level 1 vs. 3	< 0.001	1.170
Level 3	155.0 (91.0)	0.633 [0.437 , 0.771] n = 37, (d)			60.6	167.9			Level 2 vs. 3	< 0.001	0.990
RMSSD [ms]			0.8	0.386 n = 36			N/A [g]	N/A [g]	comparison:	p-value:	effect size:
Level 1	2.5 (6.3)	0.031 [-0.238 , 0.296] n = 38, (d)			98.4	272.9			N/A [g]	N/A [g]	N/A [g]
Level 2	2.4 (7.1)	0.108 [-0.164 , 0.364] n = 38, (d)			94.4	261.8			N/A [g]	N/A [g]	N/A [g]
Level 3	2.8 (8.7)	0.148 [-0.128 , 0.403] n = 37, (d)			92.3	255.9			N/A [g]	N/A [g]	N/A [g]
HF [ms^2]			0.3	0.579 n = 36			N/A [g]	N/A [g]	comparison:	p-value:	effect size:
Level 1	14.0 (73.5)	0.000 [-0.267 , 0.267] n = 38, (d)			100.0	277.2			N/A [g]	N/A [g]	N/A [g]
Level 2	11.5 (78.0)	0.151 [-0.121 , 0.402] n = 38, (d)			92.1	255.4			N/A [g]	N/A [g]	N/A [g]
Level 3	15.0 (71.0)	0.176 [-0.099 , 0.427] n = 37, (d)			90.8	251.6			N/A [g]	N/A [g]	N/A [g]
HFnu [nu]			0.3	0.571 n = 36			N/A [g]	N/A [g]	comparison:	p-value:	effect size:
Level 1	10.0 ± 22.4	0.443 [0.200 , 0.635] n = 38, (d)			74.6	206.9			N/A [g]	N/A [g]	N/A [g]
Level 2	11.9 ± 22.4	0.417 [0.169 , 0.616] n = 38, (d)			76.4	211.6			N/A [g]	N/A [g]	N/A [g]
Level 3	7.9 ± 22.0	0.471 [0.229 , 0.658] n = 37, (d)			72.7	201.6			N/A [g]	N/A [g]	N/A [g]
SD1 [ms]			0.4	0.533 n = 36			N/A [g]	N/A [g]	comparison:	p-value:	effect size:
Level 1	1.8 (4.5)	0.033 [-0.236 , 0.298] n = 38, (d)			98.3	272.6			N/A [g]	N/A [g]	N/A [g]
Level 2	1.6 (4.9)	0.131 [-0.142 , 0.384] n = 38, (d)			93.2	258.4			N/A [g]	N/A [g]	N/A [g]
Level 3	1.8 (6.1)	0.163 [-0.113 , 0.416] n = 37, (d)			91.5	253.6			N/A [g]	N/A [g]	N/A [g]
PNSIndex []			0.9	0.345 n = 36			N/A [g]	N/A [g]	comparison:	p-value:	effect size:

Experiment Phase 2

HRV Parameter	Descriptives[a] mean ± SD / median (IQR)	Relative Reliability ICC$_{3,1}$[b] 95% CI	F[c]	p[d]	Validity F[e]	p[f]	post-hoc tests			Absolute Reliability SEM %	SDD %
mRR [ms]			0.9	0.338 n = 22	25.1	< 0.001	comparison:	p-value:	effect size:		
Level 1	270.0 (132.5)	0.806 [0.677 , 0.886] n = 34, (d)					Level 1 vs. 2	< 0.001	0.849	44.0	122.1
Level 2	287.5 (131.5)	0.829 [0.711 , 0.901] n = 33, (d)					Level 1 vs. 3	< 0.001	1.227	41.4	114.6
Level 3	221.0 (172.5)	0.856 [0.754 , 0.917] n = 33, (d)					Level 2 vs. 3	< 0.001	0.933	37.9	105.2
RMSSD [ms]			1.5	0.221 n = 22	N/A [g]	N/A [g]	comparison:	p-value:	effect size:		
Level 1	8.6 (9.3)	0.490 [0.241 , 0.678] n = 34, (d)					N/A [g]	N/A [g]	N/A [g]	71.4	198.0
Level 2	8.6 (10.1)	0.690 [0.503 , 0.815] n = 33, (d)					N/A [g]	N/A [g]	N/A [g]	55.7	154.3
Level 3	4.6 (12.0)	0.503 [0.252 , 0.690] n = 33, (d)					N/A [g]	N/A [g]	N/A [g]	70.5	195.4
HF [ms^2]			1.5	0.225 n = 22	N/A [g]	N/A [g]	comparison:	p-value:	effect size:		
Level 1	67.0 (122.0)	0.427 [0.164 , 0.633] n = 34, (d)					N/A [g]	N/A [g]	N/A [g]	75.7	209.8
Level 2	60.0 (103.0)	0.582 [0.355 , 0.745] n = 33, (d)					N/A [g]	N/A [g]	N/A [g]	64.7	179.2
Level 3	27.0 (125.5)	0.509 [0.260 , 0.694] n = 33, (d)					N/A [g]	N/A [g]	N/A [g]	70.1	194.3
HFnu [nu]			0.4	0.350 n = 22	N/A [g]	N/A [g]	comparison:	p-value:	effect size:		
Level 1	3.4 ± 24.7	0.003 [-0.279 , 0.286] n = 34, (d)					N/A [g]	N/A [g]	N/A [g]	99.8	276.8
Level 2	5.0 ± 25.2	0.048 [-0.242 , 0.330] n = 33, (d)					N/A [g]	N/A [g]	N/A [g]	97.6	270.5
Level 3	6.2 ± 23.8	0.467 [0.208 , 0.665] n = 33, (d)					N/A [g]	N/A [g]	N/A [g]	73.0	202.4
SD1 [ms]			2.0	0.212 n = 22	N/A [g]	N/A [g]	comparison:	p-value:	effect size:		
Level 1	6.1 (6.5)	0.487 [0.237 , 0.677] n = 34, (d)					N/A [g]	N/A [g]	N/A [g]	71.6	198.5
Level 2	6.1 (6.6)	0.687 [0.499 , 0.814] n = 33, (d)					N/A [g]	N/A [g]	N/A [g]	55.9	155.1
Level 3	3.3 (8.5)	0.502 [0.252 , 0.690] n = 33, (d)					N/A [g]	N/A [g]	N/A [g]	70.6	195.6
PNSIndex []			0.1	0.754 n = 22	20.2	< 0.001	comparison:	p-value:	effect size:		

	HRV Parameter	Phase 1 Descriptives	Phase 1 ICC$_{3,1}$ [95% CI]	n	SEM %	SDD %	comparison	p-value	effect size	Phase 2 Descriptives	Phase 2 ICC$_{3,1}$ 95% CI	n	comparison	p-value	effect size	SEM %	SDD %
51	Level 1	0.6 (0.6)	0.477 [0.240 , 0.660]	n = 38, (d)	72.3	200.5	N/A (g)	N/A (g)	N/A (g)	1.8 (0.9)	0.698 [0.518 , 0.819]	n = 34, (d)	Level 1 vs. 2	< 0.001	0.728	55.0	152.3
	Level 2	0.7 (0.5)	0.414 [0.165 , 0.614]	n = 38, (d)	76.6	212.2	N/A (g)	N/A (g)	N/A (g)	1.9 (0.8)	0.696 [0.511 , 0.819]	n = 33, (d)	Level 1 vs. 3	< 0.001	1.134	55.1	152.8
	Level 3	0.8 (0.6)	0.473 [0.231 , 0.659]	n = 37, (d)	72.6	201.2	N/A (g)	N/A (g)	N/A (g)	1.3 (1.3)	0.754 [0.596 , 0.856]	n = 33, (d)	Level 2 vs. 3	< 0.001	0.833	49.6	137.5

Continued

Game: Simon

Experiment Phase 1

	HRV Parameter	Descriptives(a) mean ± SD / median (IQR)	Relative Reliability ICC$_{3,1}$ [95% CI]	n	F(b)	p(c)	n	Absolute Reliability SEM %	SDD %	Validity F(e)	p(f)	post-hoc tests		
52	mRR [ms]				0.0	0.883	n = 33			N/A (g)	N/A (g)	comparison:	p-value:	effect size:
	Level 1	141.6 ± 64.6	0.501 [0.263 , 0.682]	n = 36, (d)				70.6	195.8			N/A (g)	N/A (g)	N/A (g)
	Level 2	154.0 (96.0)	0.194 [-0.089 , 0.449]	n = 35, (d)				89.8	248.9			N/A (g)	N/A (g)	N/A (g)
	Level 3	134.0 (107.5)	0.622 [0.423 , 0.764]	n = 37, (d)				61.5	170.4			N/A (g)	N/A (g)	N/A (g)
	RMSSD [ms]				0.4	0.532	n = 33			N/A (g)	N/A (g)	comparison:	p-value:	effect size:
	Level 1	3.3 (7.4)	0.041 [-0.236 , 0.312]	n = 36, (d)				97.9	271.4			N/A (g)	N/A (g)	N/A (g)
	Level 2	2.9 (7.3)	0.044 [-0.237 , 0.319]	n = 35, (d)				97.8	271.0			N/A (g)	N/A (g)	N/A (g)
	Level 3	1.3 (7.7)	0.001 [-0.270 , 0.272]	n = 37, (d)				99.9	277.0			N/A (g)	N/A (g)	N/A (g)
	HF [ms^2]				3.8	0.053	n = 33			N/A (g)	N/A (g)	comparison:	p-value:	effect size:
	Level 1	14.5 (77.0)	0.002 [-0.273 , 0.276]	n = 36, (d)				99.9	276.9			N/A (g)	N/A (g)	N/A (g)
	Level 2	1.0 (96.0)	0.000 [-0.279 , 0.279]	n = 35, (d)				100.0	277.2			N/A (g)	N/A (g)	N/A (g)
	Level 3	9.0 (79.3)	0.000 [-0.271 , 0.271]	n = 37, (d)				100.0	277.2			N/A (g)	N/A (g)	N/A (g)
	HFnu [nu]				0.1	0.821	n = 33			N/A (g)	N/A (g)	comparison:	p-value:	effect size:
	Level 1	9.2 ± 23.6	0.052 [-0.226 , 0.322]	n = 36, (d)				97.4	269.9			N/A (g)	N/A (g)	N/A (g)
	Level 2	11.1 ± 30.1	0.535 [0.301 , 0.708]	n = 35, (d)				68.2	189.0			N/A (g)	N/A (g)	N/A (g)
	Level 3	13.3 ± 20.8	0.302 [0.034 , 0.530]	n = 37, (d)				83.5	231.6			N/A (g)	N/A (g)	N/A (g)
	SD1 [ms]				0.2	0.636	n = 33			N/A (g)	N/A (g)	comparison:	p-value:	effect size:
	Level 1	2.3 (5.3)	0.045 [-0.233 , 0.315]	n = 36, (d)				97.7	270.9			N/A (g)	N/A (g)	N/A (g)
	Level 2	2.0 (5.1)	0.048 [-0.233 , 0.322]	n = 35, (d)				97.6	270.5			N/A (g)	N/A (g)	N/A (g)
	Level 3	0.9 (5.5)	0.001 [-0.270 , 0.272]	n = 37, (d)				99.5	277.0			N/A (g)	N/A (g)	N/A (g)
	PNSIndex []				0.1	0.761	n = 33			N/A (g)	N/A (g)	comparison:	p-value:	effect size:
	Level 1	0.6 (0.6)	0.351 [0.084 , 0.571]	n = 36, (d)				80.6	223.3			N/A (g)	N/A (g)	N/A (g)
	Level 2	0.8 (0.7)	0.313 [0.038 , 0.544]	n = 35, (d)				82.9	229.7			N/A (g)	N/A (g)	N/A (g)
	Level 3	0.7 (0.8)	0.422 [0.171 , 0.622]	n = 37, (d)				76.0	210.7			N/A (g)	N/A (g)	N/A (g)

Experiment Phase 2

HRV Parameter	Descriptives(a) mean ± SD / median (IQR)	Relative Reliability ICC$_{3,1}$(b) 95% CI	n	F(c)	p(d)	n	Validity F(e)	p(f)	post-hoc tests			Absolute Reliability SEM %	SDD %
mRR [ms]				0.9	0.339	n = 22	0.1	0.9	comparison:	p-value:	effect size:		
Level 1	291.0 (158.0)	0.847 [0.735 , 0.914]	n = 31, (d)						Level 1 vs. 2	N/A (h)	N/A (h)	39.1	108.4
Level 2	302.5 (152.5)	0.825 [0.707 , 0.898]	n = 34, (d)						Level 1 vs. 3	N/A (h)	N/A (h)	41.8	116.0
Level 3	207.5 (168.3)	0.616 [0.400 , 0.768]	n = 33, (d)						Level 2 vs. 3	N/A (h)	N/A (h)	62.0	171.8
RMSSD [ms]				0.0	0.898	n = 22	N/A (g)	N/A (g)	comparison:	p-value:	effect size:		
Level 1	9.7 (11.1)	0.503 [0.244 , 0.696]	n = 31, (d)						N/A (g)	N/A (g)	N/A (g)	70.5	195.4
Level 2	8.9 (10.5)	0.479 [0.228 , 0.671]	n = 34, (d)						N/A (g)	N/A (g)	N/A (g)	72.2	200.1
Level 3	4.5 (12.3)	0.531 [0.289 , 0.710]	n = 33, (d)						N/A (g)	N/A (g)	N/A (g)	68.5	189.8
HF [ms^2]				0.3	0.568	n = 22	N/A (g)	N/A (g)	comparison:	p-value:	effect size:		
Level 1	72.0 (129.0)	0.447 [0.174 , 0.656]	n = 31, (d)						N/A (g)	N/A (g)	N/A (g)	74.4	206.1
Level 2	70.5 (130.5)	0.449 [0.191 , 0.650]	n = 34, (d)						N/A (g)	N/A (g)	N/A (g)	74.2	205.8
Level 3	29.0 (98.8)	0.524 [0.279 , 0.705]	n = 33, (d)						N/A (g)	N/A (g)	N/A (g)	69.0	191.2
HFnu [nu]				0.0	0.839	n = 22	N/A (g)	N/A (g)	comparison:	p-value:	effect size:		
Level 1	4.2 ± 31.1	0.000 [-0.296 , 0.296]	n = 31, (d)						N/A (g)	N/A (g)	N/A (g)	100.0	277.2
Level 2	2.6 ± 31.1	0.000 [-0.283 , 0.283]	n = 34, (d)						N/A (g)	N/A (g)	N/A (g)	100.0	277.2
Level 3	9.7 ± 31.1	0.000 [-0.287 , 0.287]	n = 33, (d)						N/A (g)	N/A (g)	N/A (g)	100.0	277.2
SD1 [ms]				0.1	0.823	n = 22	N/A (g)	N/A (g)	comparison:	p-value:	effect size:		
Level 1	6.8 (7.7)	0.505 [0.246 , 0.697]	n = 31, (d)						N/A (g)	N/A (g)	N/A (g)	70.4	195.0
Level 2	6.4 (7.4)	0.480 [0.229 , 0.672]	n = 34, (d)						N/A (g)	N/A (g)	N/A (g)	72.1	199.9
Level 3	3.1 (8.6)	0.534 [0.292 , 0.712]	n = 33, (d)						N/A (g)	N/A (g)	N/A (g)	68.3	189.2
PNSIndex []				0.2	0.692	n = 22	1.2	0.3	comparison:	p-value:	effect size:		
Level 1	1.8 (1.2)	0.684 [0.486 , 0.815]	n = 31, (d)						Level 1 vs. 2	N/A (h)	N/A (h)	56.2	155.8
Level 2	1.8 (1.2)	0.666 [0.472 , 0.798]	n = 34, (d)						Level 1 vs. 3	N/A (h)	N/A (h)	57.8	160.2
Level 3	1.2 (1.3)	0.771 [0.622 , 0.867]	n = 33, (d)						Level 2 vs. 3	N/A (h)	N/A (h)	47.9	132.6

Continued

Game: Habitats

Experiment Phase 1

HRV Parameter	Descriptives(a) mean ± SD / median (IQR)	Relative Reliability ICC$_{3,1}$ [95% CI]	n	F(b)	p(c)	n	Absolute Reliability SEM %	SDD %	Validity F(e)	p(f)	post-hoc tests		
mRR [ms]				0.0	0.923	n = 30			0.1	0.917	comparison:	p-value:	effect size:
Level 1	126.3 ± 58.2	0.548 [0.322 , 0.715]	n = 36, (d)				67.2	186.4			Level 1 vs. 2	N/A (h)	N/A (h)
Level 2	128.5 ± 55.8	0.545 [0.310 , 0.717]	n = 34, (d)				67.5	187.0			Level 1 vs. 3	N/A (h)	N/A (h)
Level 3	137.3 ± 55.1	0.525 [0.293 , 0.699]	n = 36, (d)				68.9	191.0			Level 2 vs. 3	N/A (h)	N/A (h)
RMSSD [ms]				1.0	0.307	n = 30			N/A (g)	N/A (g)	comparison:	p-value:	effect size:
Level 1	0.2 (7.2)	0.027 [-0.249 , 0.299]	n = 36, (d)				98.6	273.4			N/A (g)	N/A (g)	N/A (g)
Level 2	0.6 (7.1)	0.000 [-0.283 , 0.283]	n = 34, (d)				100.0	277.2			N/A (g)	N/A (g)	N/A (g)
Level 3	1.0 (7.6)	0.007 [-0.268 , 0.281]	n = 36, (d)				99.6	276.2			N/A (g)	N/A (g)	N/A (g)
HF [ms^2]				0.9	0.350	n = 30			N/A (g)	N/A (g)	comparison:	p-value:	effect size:
Level 1	3.5 (88.8)	0.096 [-0.183 , 0.361]	n = 36, (d)				95.1	263.5			N/A (g)	N/A (g)	N/A (g)
Level 2	8.5 (60.0)	0.000 [-0.283 , 0.283]	n = 34, (d)				100.0	277.2			N/A (g)	N/A (g)	N/A (g)
Level 3	8.5 (80.3)	0.000 [-0.275 , 0.275]	n = 36, (d)				100.0	277.2			N/A (g)	N/A (g)	N/A (g)
HFnu [nu]				0.4	0.554	n = 30			N/A (g)	N/A (g)	comparison:	p-value:	effect size:
Level 1	7.8 ± 26.4	0.237 [-0.040 , 0.481]	n = 36, (d)				87.3	242.1			N/A (g)	N/A (g)	N/A (g)
Level 2	0.9 ± 24.4	0.241 [-0.045 , 0.490]	n = 34, (d)				87.1	241.5			N/A (g)	N/A (g)	N/A (g)
Level 3	3.5 ± 24.0	0.262 [-0.014 , 0.501]	n = 36, (d)				85.9	238.1			N/A (g)	N/A (g)	N/A (g)
SD1 [ms]				0.6	0.423	n = 30			N/A (g)	N/A (g)	comparison:	p-value:	effect size:
Level 1	0.0 (5.1)	0.039 [-0.238 , 0.310]	n = 36, (d)				98.0	271.7			N/A (g)	N/A (g)	N/A (g)
Level 2	0.4 (5.0)	0.000 [-0.283 , 0.283]	n = 34, (d)				100.0	277.2			N/A (g)	N/A (g)	N/A (g)
Level 3	0.8 (5.4)	0.003 [-0.272 , 0.277]	n = 36, (d)				99.8	276.8			N/A (g)	N/A (g)	N/A (g)
PNSIndex []				4.3	0.038	n = 30			N/A (g)	N/A (g)	comparison:	p-value:	effect size:
Level 1	0.5 (0.6)	0.482 [0.239 , 0.668]	n = 36, (d)				72.0	199.5			N/A (g)	N/A (g)	N/A (g)
Level 2	0.6 (0.6)	0.479 [0.227 , 0.671]	n = 34, (d)				72.2	200.1			N/A (g)	N/A (g)	N/A (g)
Level 3	0.6 (0.6)	0.449 [0.199 , 0.644]	n = 36, (d)				74.2	205.8			N/A (g)	N/A (g)	N/A (g)

Experiment Phase 2

HRV Parameter	Descriptives(a) mean ± SD / median (IQR)	Relative Reliability ICC$_{3,1}$(b) 95% CI	n	F(c)	p(d)	n	Validity F(e)	p(f)	post-hoc tests			Absolute Reliability SEM %	SDD %
mRR [ms]				2.4	0.120	n = 26	2.7	0.1	comparison:	p-value:	effect size:		
Level 1	280.0 (165.0)	0.842 [0.737 , 0.907]	n = 36, (d)						Level 1 vs. 2	N/A (h)	N/A (h)	39.7	110.2
Level 2	278.0 (154.5)	0.832 [0.720 , 0.901]	n = 35, (d)						Level 1 vs. 3	N/A (h)	N/A (h)	41.0	113.6
Level 3	197.0 (138.5)	0.843 [0.735 , 0.909]	n = 34, (d)						Level 2 vs. 3	N/A (h)	N/A (h)	39.6	109.8
RMSSD [ms]				0.2	0.694	n = 26	N/A (g)	N/A (g)	comparison:	p-value:	effect size:		
Level 1	8.8 (11.6)	0.357 [0.092 , 0.575]	n = 36, (d)						N/A (g)	N/A (g)	N/A (g)	80.2	222.3
Level 2	9.0 (10.8)	0.286 [0.008 , 0.523]	n = 35, (d)						N/A (g)	N/A (g)	N/A (g)	84.5	234.2
Level 3	4.5 (9.7)	0.562 [0.332 , 0.729]	n = 34, (d)						N/A (g)	N/A (g)	N/A (g)	66.2	183.4
HF [ms^2]				0.0	0.928	n = 26	N/A (g)	N/A (g)	comparison:	p-value:	effect size:		
Level 1	69.0 (115.0)	0.445 [0.194 , 0.641]	n = 36, (d)						N/A (g)	N/A (g)	N/A (g)	74.5	206.5
Level 2	70.0 (112.8)	0.434 [0.177 , 0.636]	n = 35, (d)						N/A (g)	N/A (g)	N/A (g)	75.2	208.5
Level 3	28.0 (101.5)	0.459 [0.202 , 0.656]	n = 34, (d)						N/A (g)	N/A (g)	N/A (g)	73.6	203.9
HFnu [nu]				1.4	0.240	n = 26	N/A (g)	N/A (g)	comparison:	p-value:	effect size:		
Level 1	3.7 ± 26.6	0.213 [-0.065 , 0.461]	n = 36, (d)						N/A (g)	N/A (g)	N/A (g)	88.7	245.9
Level 2	3.8 ± 28.0	0.173 [-0.111 , 0.431]	n = 35, (d)						N/A (g)	N/A (g)	N/A (g)	90.9	252.1
Level 3	2.3 ± 28.0	0.455 [0.198 , 0.654]	n = 34, (d)						N/A (g)	N/A (g)	N/A (g)	73.8	204.6
SD1 [ms]				0.2	0.688	n = 26	N/A (g)	N/A (g)	comparison:	p-value:	effect size:		
Level 1	6.1 (7.9)	0.354 [0.088 , 0.573]	n = 36, (d)						N/A (g)	N/A (g)	N/A (g)	80.4	222.8
Level 2	6.3 (7.7)	0.282 [0.004 , 0.520]	n = 35, (d)						N/A (g)	N/A (g)	N/A (g)	84.7	234.9
Level 3	3.1 (6.9)	0.561 [0.331 , 0.728]	n = 34, (d)						N/A (g)	N/A (g)	N/A (g)	66.3	183.7
PNSIndex []				0.4	0.542	n = 26	2.2	0.1	comparison:	p-value:	effect size:		
Level 1	1.8 (1.1)	0.742 [0.586 , 0.844]	n = 36, (d)						Level 1 vs. 2	N/A (h)	N/A (h)	50.8	140.8
Level 2	1.7 (1.2)	0.678 [0.492 , 0.805]	n = 35, (d)						Level 1 vs. 3	N/A (h)	N/A (h)	56.7	157.3
Level 3	1.1 (1.1)	0.719 [0.547 , 0.832]	n = 34, (d)						Level 2 vs. 3	N/A (h)	N/A (h)	53.0	146.9

Presented are descrpitve statistics, ICCs, F-values, p-values, SEM%, and SDD%, of the test and the retest data in Experiment Phase 1 and 2, respectively.

(a) descriptive statistics; data is reported as mean ± standard deviation for continuous parametric data and median (interquartile range) for continuous non-parametric data
(b) F-value for the main effect of timepoint (test- vs. rerest-measurement) from the two-way repeated measures ANOVA.
(c) p-value for the main effect of timepoint (test- vs. rerest-measurement) from the two-way repeated measures ANOVA.
(d) missing data due to low quality data (≥ 5 % of beats corrected by the automatic beat correction algorithm of Kubios HRV Premium) that was excluded from analysis
(e) F-value for the main effect of level from the two-way repeated measures ANOVA.
(f) p-value for the main effect of level from the two-way repeated measures ANOVA.
(g) not applicable, because criteria for analysis were not met. In particular: Only outcome measures with at least a fair test-retest reliability (i.e., ICC3,1 ≥ 0.5) in all three levels of standardized task demands (i.e., Level 1 = easy, Level 2 = challenging, and Level 3 = excessive) were co
(h) not applicable, because main effect is not significant => no pairwise post-hoc comparisons

ICC ≥ 0.9: excellent
0.75 ≤ ICC < 0.9: good
0.5 ≤ ICC < 0.75: fair
ICC < 0.50: poor

Abbreviations: mRR, mean R-R time interval; RMSSD, root mean square of sccessive RR interval differences; HF, absolute power of the high-frequency (0.15 – 0.4 Hz) band; SD1, Poincaré plot standard deviation perpendicular to the line of identity; PNSIndex, parasympathetic nervous system tone index.

References

1. Kottner J, et al. Guidelines for Reporting Reliability and Agreement Studies (GRRAS) were proposed. International Journal of Nursing Studies. 2011;48(6):661-671. doi: https://doi.org/10.1016/j.ijnurstu.2011.01.016.

2. von Elm E, et al. The Strengthening the Reporting of Observational Studies in Epidemiology (STROBE) statement: guidelines for reporting observational studies. The Lancet. 2007;370(9596):1453-1457. doi: https://doi.org/10.1016/S0140-6736(07)61602-X.
